# *EIF4G1* and *RAN* as Possible Drivers for Malignant Pleural Mesothelioma

**DOI:** 10.3390/ijms21144856

**Published:** 2020-07-09

**Authors:** Irene Dell’Anno, Marcella Barbarino, Elisa Barone, Antonio Giordano, Luca Luzzi, Maria Bottaro, Loredana Migliore, Silvia Agostini, Alessandra Melani, Ombretta Melaiu, Calogerina Catalano, Monica Cipollini, Roberto Silvestri, Alda Corrado, Federica Gemignani, Stefano Landi

**Affiliations:** 1 Department of Biology, Genetic Unit, University of Pisa, 56126 Pisa, Italy; irene.dellanno@biologia.unipi.it (I.D.); elisa_barone@ymail.com (E.B.); loredana.migliore@student.unisi.it (L.M.); silvietta.agostini@gmail.com (S.A.); alessandra-29@hotmail.it (A.M.); ombretta.melaiu@unipi.it (O.M.); cal.catalano@gmail.com (C.C.); monica.cipollini@unipi.it (M.C.); r.silvestri17@gmail.com (R.S.); corradoalda@gmail.com (A.C.); federica.gemignani@unipi.it (F.G.); 2 Department of Medical Biotechnologies, University of Siena, 53100 Siena, Italy; marcella.barbarino@unisi.it (M.B.); president@shro.org (A.G.); mariaeusebia.bottaro@gmail.com (M.B.); 3 Sbarro Institute for Cancer Research and Molecular Medicine, Center for Biotechnology, College of Science and Technology, Temple University, Philadelphia, PA 19122, USA; 4 Department of Medicine, Surgery and Neurosciences, Siena University Hospital, 53100 Siena, Italy; dr.luca.luzzi@gmail.com; 5 Immuno-Oncology Laboratory, Department of Paediatric Haematology/Oncology and of Cell and Gene Therapy, Ospedale Pediatrico Bambino Gesù, IRCCS, 00165 Rome, Italy; 6 Department of Internal Medicine V, University of Heidelberg, 69117 Heidelberg, Germany; 7 Department of Bioscience, University of Milan, 20133 Milan, Italy

**Keywords:** cancer driving gene, EIF4G1, MPM, RAN, siRNA, importazole

## Abstract

For malignant pleural mesothelioma (MPM) novel therapeutic strategies are urgently needed. In a previous study, we identified 51 putative cancer genes over-expressed in MPM tissues and cell lines. Here, we deepened the study on nine of them (*ASS1*, *EIF4G1*, *GALNT7*, *GLUT1*, *IGF2BP3* (*IMP3*), *ITGA4*, *RAN*, *SOD1*, and *THBS2*) to ascertain whether they are truly mesothelial cancer driver genes (CDGs) or genes overexpressed in an adaptive response to the tumoral progression (“passenger genes”). Through a fast siRNA-based screening, we evaluated the consequences of gene depletion on migration, proliferation, colony formation capabilities, and caspase activities of four MPM (Mero-14, Mero-25, IST-Mes2, and NCI-H28) and one SV40-immortalized mesothelial cell line (MeT-5A) as a non-malignant model. The depletion of *EIF4G1* and *RAN* significantly reduced cell proliferation and colony formation and increased caspase activity. In particular, the findings for RAN resemble those observed for other types of cancer. Thus, we evaluated the in vitro effects of importazole (IPZ), a small molecule inhibitor of the interaction between RAN and importin-β. We showed that IPZ could have effects similar to those observed following *RAN* gene silencing. We also found that primary cell lines from one out of three MPM patients were sensitive to IPZ. As *EIF4G1* and *RAN* deserve further investigation with additional in vitro and in vivo studies, they emerged as promising CDGs, suggesting that their upregulation could play a role in mesothelial tumorigenesis and aggressiveness. Furthermore, present data propose the molecular pathways dependent on RAN as a putative pharmacological target for MPM patients in the view of a future personalized medicine.

## 1. Introduction

Malignant pleural mesothelioma (MPM) is a rare occupational disease mainly due to asbestos handling [1,2,3,4]. To date, novel diagnostic and therapeutic targets for MPM are urgently needed. For this purpose, in the recent past we undertook research for identifying mesothelial cancer driving genes (CDGs), since the deregulation of normal transcription levels is one of the mechanisms driving tumorigenesis. However, gene deregulation could also be an epiphenomenon, i.e., the consequence of phenotypic alterations not truly driving the process of tumorigenesis (these deregulated genes could be defined as “passenger genes” (PGs)). Therefore, the study of the cancer transcriptomic landscape is a good starting point for further studies aimed at discriminating CDGs among a plethora of PGs.

In a previous work, we carried out a broad literature search of transcriptomic studies and identified 119 genes deregulated in MPM [5]. In order to confirm the status of these genes, we performed a preliminary screening by comparing their expression in 15 surgically resected MPMs and in 20 non-malignant pleura tissues. We found that in MPM, the transcripts of 51 genes were over-expressed in a statistically significant way when compared to non-MPM tissues, narrowing the list of candidate CDGs [6]. As a further criterion for discriminating CDGs from PGs, in the same study we analyzed the mRNA expression of these 51 genes on two MPM cell lines, ending with a short list of 28 genes. 

In the present work, we evaluated whether *ASS1*, *EIF4G1*, *GALNT7*, *GLUT1*, *IGF2BP3* (*IMP3*), *ITGA4*, *RAN*, *SOD1*, and *THBS2* could be *bone fide* CDGs of pleural tumorigenesis. They were selected because of the poor or lack of knowledge in the context of MPM despite a body of literature supporting their role in cancer. These genes are representative of pathways deregulated in tumorigenesis such as arginine metabolism (*ASS1*) [7], protein translation (*EIF4G1*) [8] or protein glycosylation and N-acetylgalactosamination (*GALNT7*) [9], glycolysis (*GLUT1*) [10], protein–RNA complex formation (*IGF2BP3*) [11], cell surface adhesion and signaling (*ITGA4*) [12], the RAS signaling pathway (*RAN*) [13], free superoxide radical metabolism (*SOD1*) [14], and cell-to-cell and cell-to-matrix interactions (*THBS2*) [15]. For *GLUT1* and *IGF2BP3* an increased expression in MPM was observed and a possible use as MPM biomarkers was suggested [16]. The role of *ASS1* in mesothelial tumorigenesis is subject to debate, since there are contrasting studies on tissues and 3D spheroids where ASS1 has been reported as either down-regulated or up-regulated [17,18]. 

In particular, we analyzed the migration, proliferation, colony formation capabilities, and the caspase activities on a variety of cell lines, including primary cells from cancer patients. The findings led us to take into consideration a small molecule that could constitute a hypothetical therapeutic agent for future applications in the fight against this fatal disease.

## 2. Results

In this study *ASS1*, *EIF4G1*, *GALNT7*, *GLUT1*, *IGF2BP3*, *ITGA4*, *RAN*, *SOD1*, and *THBS2* genes were assayed on Mero-14, Mero-25, IST-Mes2, and NCI-H28, and the phenotypic changes were evaluated following gene silencing depending upon the mRNA expression. MeT-5A cells were employed as reference for protein expression. GLUT1 and SOD1 proteins were expressed mainly in NCI-H28 (for GLUT1 a relative expression of 4.2-fold was measured, *p* < 0.05), whereas their relative expression was ≤1 in Mero-25, Mero-14, and IST-Mes2 (Supplementary Materials Figure S1 and Figure S2). For ITGA4, all MPM cell lines showed a relative expression ≤1 (Supplementary Materials Figure S3). In summary, although a relevant role of these proteins in MPM cannot be ruled out, we considered that their over-expression in, maximum, one MPM cell line did not constitute sufficient evidence for pointing them as true drivers of mesothelial tumorigenesis. Therefore, in the following paragraphs we will describe the main statistically significant results obtained with the phenotypic assays after gene silencing of the remaining candidate CDGs (*ASS1*, *EIF4G1*, *IGF2BP3, GALNT7, RAN*, and *THBS2*). The results not specifically described in this section are reported in Supplementary A and the supplementary figures.

*ASS1.* A complete overview of the results is reported in Supplementary A and in Supplementary Materials Figures S4–S8. In brief, siASS1-1 caused a significant reduction (MANOVA; *p* < 0.01) in the proliferation of Mero-25 (–40% at day 6, *p* < 0.001; –35% at day 8, *p* < 0.001) and IST-Mes2 cell lines (– 23% at day 6 and 8; *p* < 0.001) (Supplementary Materials Figure S6). Mero-25 (–25%, *p* = 0.0071) and IST-Mes2 (–30%, *p* = 0.0061) cell lines showed also a decreased ability in colony formation (Supplementary Materials Figure S7). No effects were seen in the MeT-5A cell line. 

**Figure 1:**
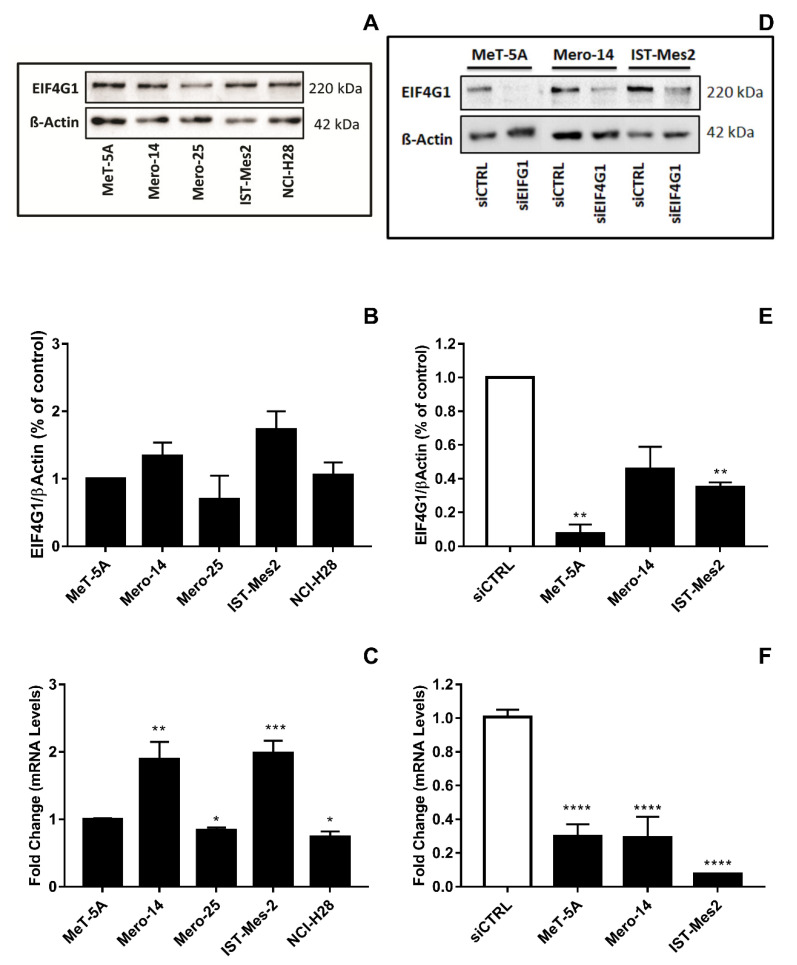
Expression of EIF4G1 in non-malignant MeT-5A and a panel of malignant pleural mesothelioma (MPM) cells, as Mero-14, Mero-25, IST-Mes2, and NCI-H28. (**A**): Picture representing basal protein levels of EIF4G1. β-Actin was used as reference. The present picture is representative of one of two experiments performed. (**B**): Histogram reporting protein levels of EIF4G1, normalized to β-actin. The histogram was generated by quantifying blots from two independent experiments and normalizing the intensity of the bands to the MeT-5A lane. (**C**): RT-qPCR showing fold changes of mRNA basal levels of *EIF4G1* gene, measured in MPM cell lines and related to MeT-5A, set to one. *RPLP0*, *HPRT1*, and *TPB* were used for normalization. (**D**): Picture representing protein levels of EIF4G1 after its depletion through siEIF4G1-1. β-Actin was used as reference. The present picture is representative of one of two experiment performed. (**E**): Histogram reporting protein levels of EIF4G1 normalized to β-actin. The histogram was generated by quantifying blots from two independent experiments and normalizing the intensity of the bands to siCTRL lane. (**F**): RT-qPCR showing, as fold change, the mRNA expression levels of *EIF4G1*, after treatment with siEIF4G1-1, related to their own siCTRL. *RPLP0*, *HPRT1*, and *TPB* were used for normalization. Error bars are SEM from three independent experiments, each performed in triplicate. Statistical significance is indicated by asterisk (*), where * *p* < 0.05; ** *p* < 0.01; *** *p* < 0.001, **** *p* < 0.0001.

**Figure 2:**
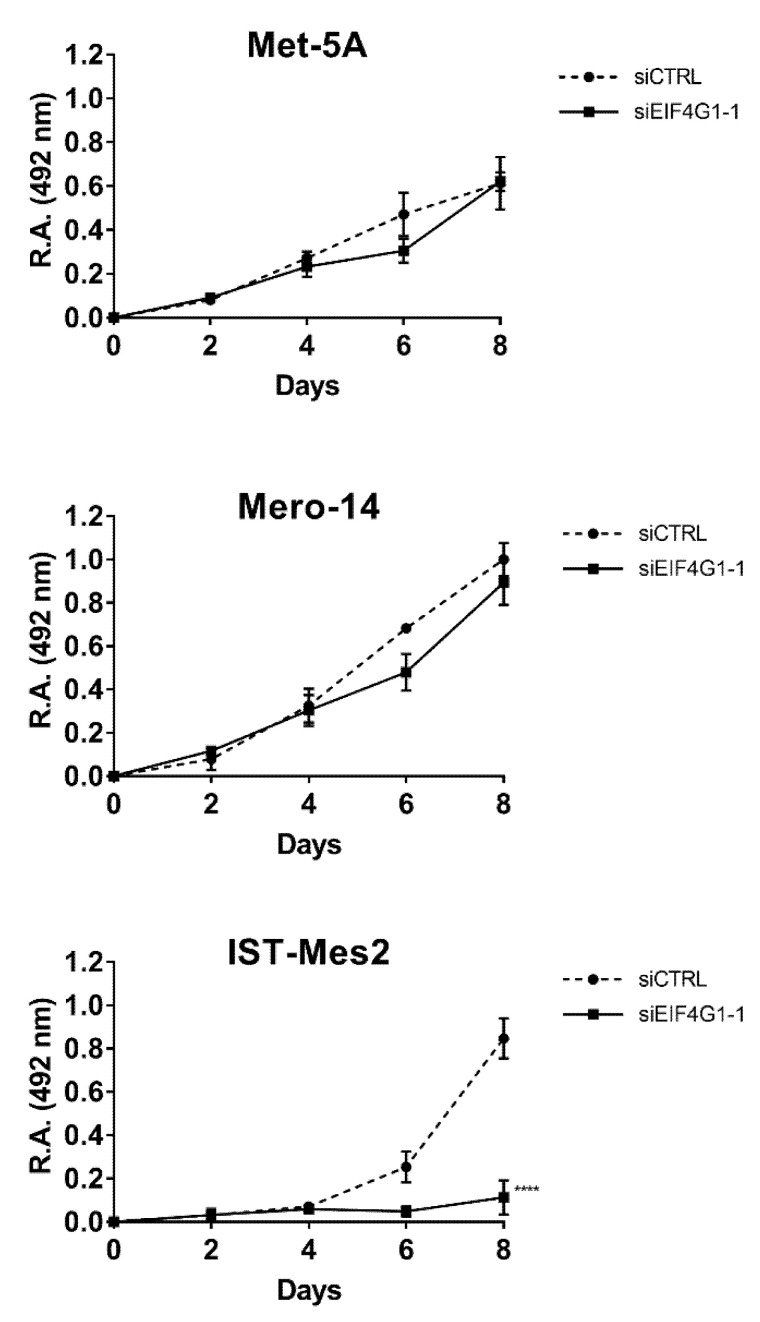
*EIF4G1* silencing and cellular growth. Proliferation curves of MeT-5A and MPM cells, as Mero-14, and IST-Mes2. The acquisition of optical density at 492 nm (relative absorbance, (R.A.)), was made every two days after the day of transfection (that is Day 0) with siCTRL or siEIF4G1-1. Error bars are SEM from three independent experiments, each performed in triplicate. Statistical significance is indicated by asterisk (*), where **** *p* < 0.0001 compared to control treatment.

**Figure 3:**
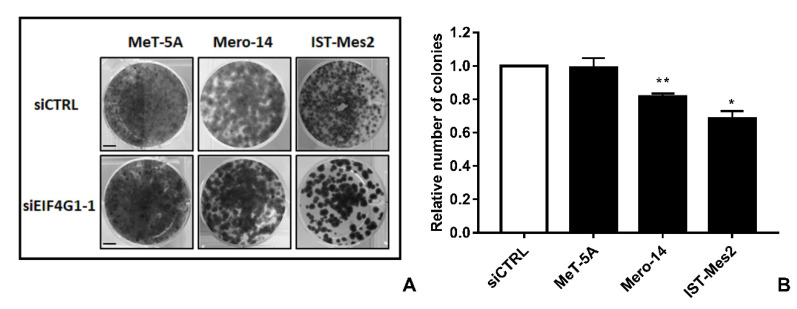
*EIF4G1* silencing and colony formation ability. (**A**): Colonies in MeT-5A and MPM cell lines fixed and stained 14 days after treatment with siCTRL or siEIF4G1-1. This is a representative picture of one experiment. Scale bar: 1 cm (**B**): Histogram represents number of colonies measured 14 days after silencing with siEIF4G1-1, compared to siCTRL. Error bars are SEM of three different experiments, each performed in triplicate. Statistical significance is indicated by asterisk (*), where * *p* < 0.05; ** *p* < 0.01; compared to control treatment.

**Figure 4:**
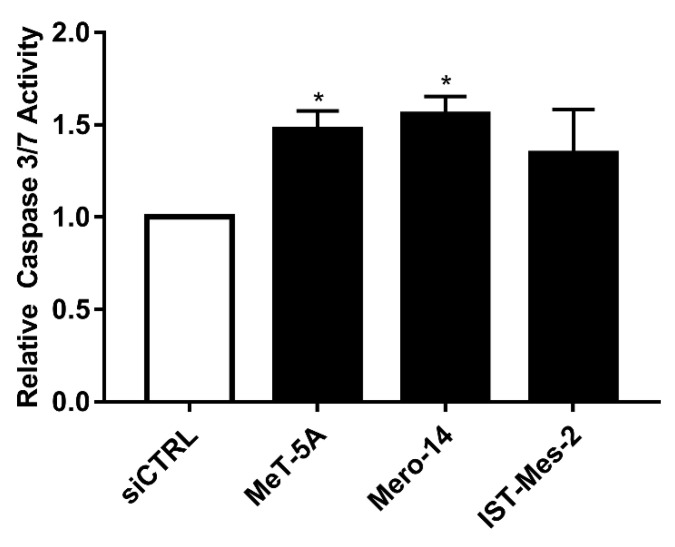
*EIF4G1* silencing and caspase -3 and 7- activities. Caspase activity measured in MeT-5A and MPM cells, after transfection with siCTRL (white bar) or siEIF4G1-1 (black bar). Error bars are the SEM of three independent experiments, each performed in triplicate. Statistical significance is indicated by asterisk (*), where * *p* < 0.05; compared to siCTRL treatment.

*EIF4G1*. Mero-14, and IST-Mes2 showed a relative expression of EIF4G1 >1 (respectively: 1.4-fold, *p* = 0.08 and 1.7-fold, *p* = 0.06) and the highest expression of mRNA (about 2-fold for both, compared to MeT-5A, *p* = 0.0045 and *p* < 0.001, respectively) (Figure 1A–C). Thus, MeT-5A, Mero-14, and IST-Mes2 were further evaluated following *EIF4G1* gene silencing. The siRNA, now on named siEIF4G1-1, was effective both at mRNA and protein level in all cell lines (Figure 1D–F). siEIF4G1-1 induced a reduction (MANOVA; *p* < 0.01) of the proliferation rate of IST-Mes2 cells (–75%, *p* < 0.001) (Figure 2). Decreased clonogenic activity was observed in all malignant cell lines, ranging from –18% in Mero-14 (*p* = 0.0088) to –32% in IST-Mes2 cells (*p* = 0.022) (Figure 3). No effects were observed in MeT-5A. *EIF4G1* depletion also caused a statistically significant increase of caspases 3 and 7 activity in all cell lines (ranging between 1.4- and 1.6-fold) with the exception of IST-Mes2 (Figure 4).

*GALNT7.* A complete overview of the results is reported in Supplementary A and in Supplementary Materials Figures S9–S12. Briefly, a significant (MANOVA; *p* < 0.01) decrease in proliferation of malignant cells was observed eight days after siGALNT7-1 (–30% in Mero-14, *p* = 0.085; –20% in IST-Mes2, *p* = 0.0035) (Supplementary Materials Figure S11). siGALNT7-1 also caused a 40% reduction in the number of colonies of IST-Mes2 cells (*p* < 0.05) (Supplementary Materials Figure S12). 

*IGF2BP3.* Most of the assays were found not affected by gene silencing of *IGF2BP3* (Supplementary A and Supplementary Materials Figures S13–S17). However, siIGF2BP3 reduced the growth (–54.6% at day 8, *p* < 0.001) (Supplementary Materials Figure S15) and the migration (–25%, *p* = 0.035) (Supplementary Materials Figure S17) of the Mero-14 cell line. 

**Figure 5:**
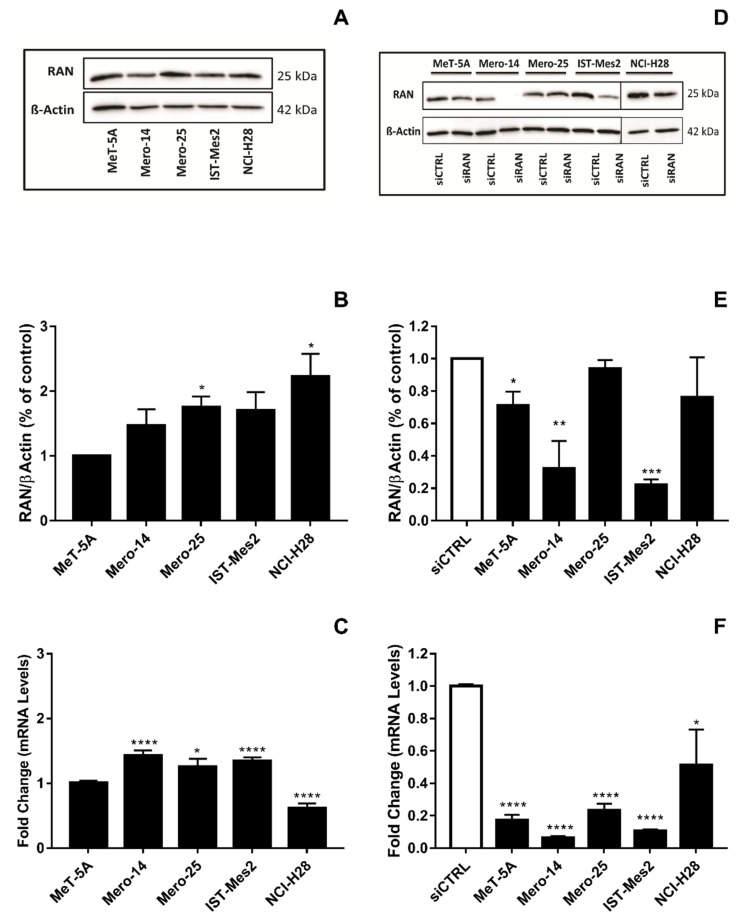
Expression of RAN in non-malignant MeT-5A and a panel of MPM cells, as Mero-14, Mero-25, IST-Mes2, and NCI-H28. (**A**): Picture representing basal protein levels of RAN. β-Actin was used as reference. The present picture is representative of one of two experiments performed. (**B**): Histogram reporting protein levels of RAN, normalized to β-actin. The histogram was generated by quantifying blots from two independent experiments and normalizing the intensity of the bands to the MeT-5A lane. (**C**): RT-qPCR showing fold changes of mRNA basal levels of *RAN*, measured in MPM cell lines and related to the MeT-5A, set to one. *RPLP0*, *HPRT1*, and *TPB* were used for normalization. (**D**): Picture representing protein levels of RAN, after its depletion through siRAN-1. β-Actin was used as reference. The present picture is representative of one of two experiment performed. The grouping of blots cropped from different parts of the same gel, or from different gels, is made explicit using a black line delineating the boundary between the gels. (**E**): Histogram reporting protein levels of RAN normalized to β-actin. The histogram was generated by quantifying blots from two independent experiments and normalizing the intensity of the bands to the siCTRL lane. (**F**): RT-qPCR showing, as fold change, the mRNA expression levels of *RAN*, after treatment with siRAN-1, related to their own siCTRL. *RPLP0*, *HPRT1*, and *TPB* were used for normalization. Error bars are SEM, from three independent experiments, each performed in triplicate. Statistical significance is indicated by asterisk (*), where * *p* < 0.05; ** *p* < 0.01; *** *p* < 0.001, **** *p* < 0.0001.

**Figure 6:**
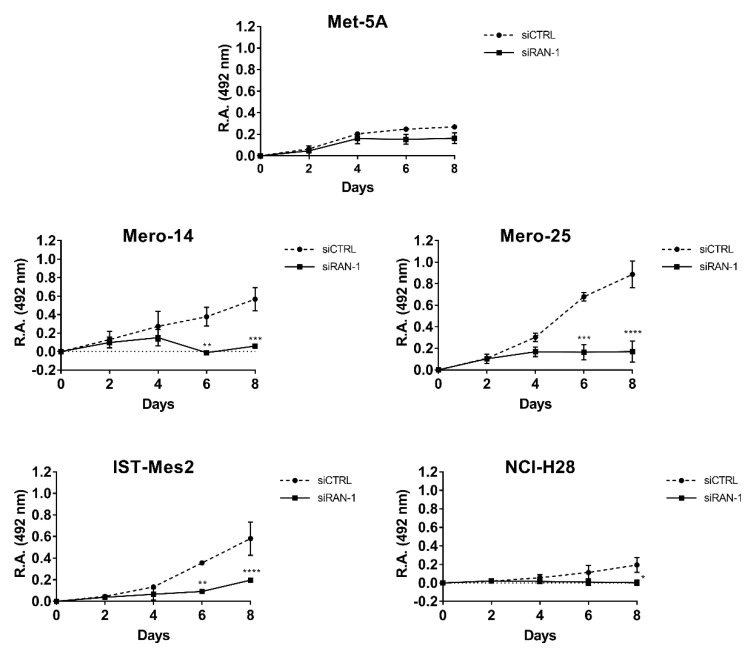
*RAN* silencing and cellular growth. SRB proliferation curves of MeT-5A, Mero-14, Mero-25, IST-Mes2, and NCI-H28. The acquisition of optical density, at 492 nm (relative absorbance, (R.A.)), was made every two days after the day of transfection (that is Day 0) with siCTRL or siRAN-1. Error bars are SEM from three independent experiments, each performed in triplicate. Statistical significance is indicated by asterisk (*), where * *p* < 0.05; ** *p* < 0.01; *** *p* < 0.001, **** *p* < 0.0001 compared to control treatment.

**Figure 7:**
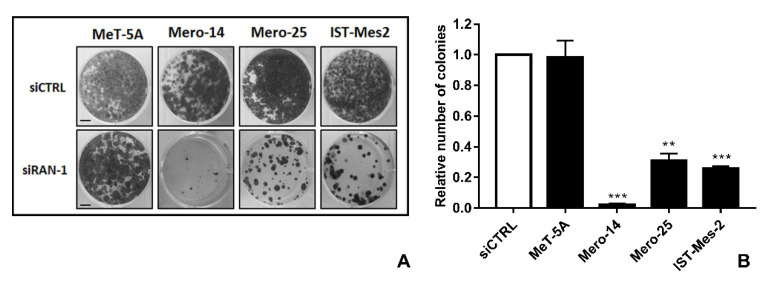
*RAN* silencing and colony formation ability. (**A**): Representative pictures of colonies in MeT-5A and MPM cell lines fixed and stained 14 days after treatment with siCTRL or siRAN-1. Scale bar: 1 cm. (**B**): Histogram represents number of colonies measured 14 days after silencing with siRAN-1, compared to siCTRL. Error bars are SEM of three different experiments, each performed in triplicate. Statistical significance is indicated by *, where ** *p* < 0.01; *** *p* < 0.001, compared to control treatment.

**Figure 8:**
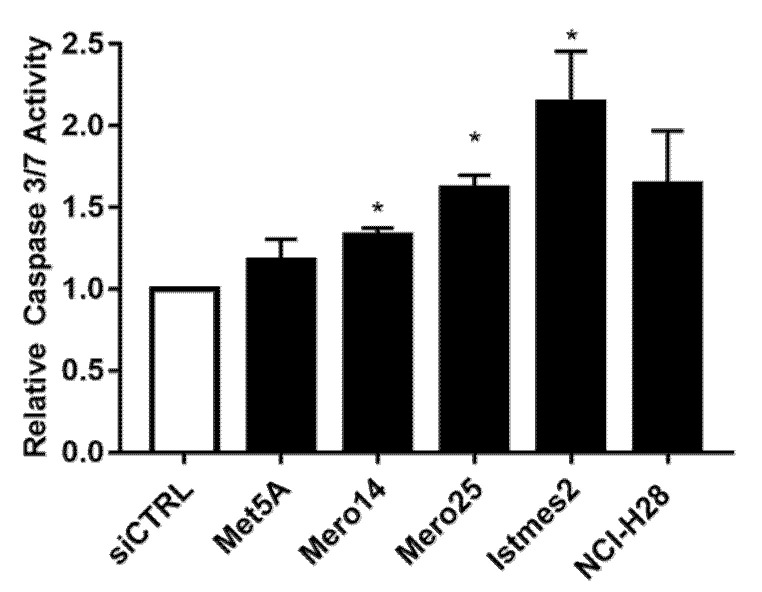
*RAN* silencing and caspase-3 and -7 activities. Caspase activity measured in MeT-5A and MPM cells, after transfection with siCTRL (white bar) or siRAN-1. Error bars are the SEM of three independent experiments, each performed in triplicate. Statistical significance is indicated by asterisk (*), where * *p* < 0.05; compared to control treatment.

*RAN.* The protein RAN showed an elevated relative expression in all MPM cell lines (Figure 5A,B). The mRNA analysis confirmed this pattern for the MPM cells, but not for NCI-H28, where mRNA basal levels resulted in a downregulation of 0.40-fold versus MeT-5A (*p* < 0.001) (Figure 5C). However, we silenced *RAN* in MeT-5A and all the MPM cells. The siRNA, from now on called siRAN-1, worked efficiently, to various extents, at both mRNA and protein levels (Figure 5D—F). Cellular growth of all malignant cell lines was clearly affected (MANOVA; *p* < 0.01) by *RAN* silencing, and this impairment was appreciable starting from the fourth day after the treatment. At day 8, the proliferation rate dropped by an average of 19.3% in NCI-H28 (*p* = 0.012), of 50% in Mero-14 and IST-Mes2 (*p* < 0.001, for both cell lines), and of 71.6% in Mero-25 (*p* < 0.001) (Figure 6). As illustrated in Figure 7, *RAN* depletion also strongly impaired the capability of colony formation of all MPM cell lines, with Mero-14 being the most affected (−98%, *p* = 0.0065) (Figure 7). Concerning the results of caspase activation, following *RAN* silencing, a statistically significant increase was observed in all MPM cell lines, ranging from 1.2- (Mero-14, *p* < 0.05) to approximately 2-fold (IST-Mes2, *p* < 0.05), with the exception of NCI-H28 (*p* = 0,18) (Figure 8). An extensive description of the results can be found in Supplementary A and Supplementary Materials Figure S18.

*THBS2.* A complete overview of the results is reported in Supplementary A and in Supplementary Materials Figures S19–S23. siTHBS2-1 treatment caused a statistically significant (MANOVA, *p* < 0.01) decrease in proliferation (at day 8, –63.6%, *p* = 0.044) (Supplementary Materials Figure S21) and colony formation activity (–70%, *p* = 0.002) of the IST-Mes2 cell line (Supplementary Materials Figure S22).

**Figure 9:**
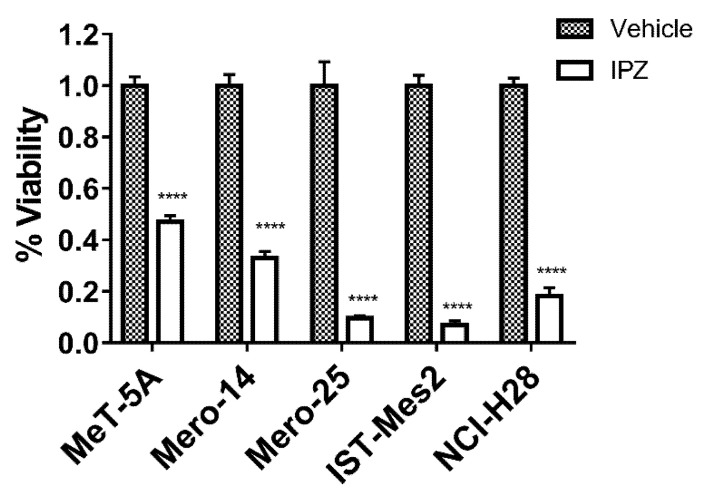
IPZ and cellular viability. Viability of MeT-5A and MPM cells when treated with the vehicle (DMSO, grey bar) or IPZ (white bar) for 72 h. Cell viability was determined using 3-(4,5-Dimethyl-2-thiazolyl)-2,5-diphenyl-2H-tetrazolium bromide (MTT) assay. Error bars are SEM from three independent experiments, each performed in triplicate. Statistical significance is indicated by asterisk (*), where **** *p* < 0.0001, compared to control treatment.

Overall, our results showed that, among all CDGs, *EIF4G1* and *RAN* were the most effective in affecting the phenotypes of MPM cell lines, thus resulting as highly likely CDGs worth of further investigation. We considered a library of molecules, but we could not find any specific inhibitor of EIF4G1 or RAN; however, we identified importazole (IPZ) as a small molecule known to hamper the RAN-dependent pathways [19,20]. This is a 2,4-diaminoquinazoline blocking the interaction between RanGTP and importin-β, thereby repressing the activity of nuclear transport. Thus, we assayed IPZ in the same cell lines reported before with the expectation of eliciting an inhibition of proliferation to a similar extent to that observed following *RAN* gene-silencing. Moreover, we assayed the cytotoxicity of IPZ in a set of primary cells from MPM patients given the known difference in drug sensitivity between primary and commercially available immortalized MPM cell lines. Cells were treated with IPZ at a range of doses (0.24, 0.48, 0.97, 1.95, 3.90, 7.81, 15.62, 31.25, 62.5, 125, 250, and 500 μM) and the calculation of the IC_50_ was performed after 24 h. The results are reported in Table 1 and showed that the IC_50_ was close to 20 µM for all cell lines, with the exception of Mero-14 (10.92 µM). Figure 9 depicts the response of cells treated with the dose IC_50_ for 72 h, showing a dramatic drop of proliferation for almost all the cell lines. 

**Figure 10:**
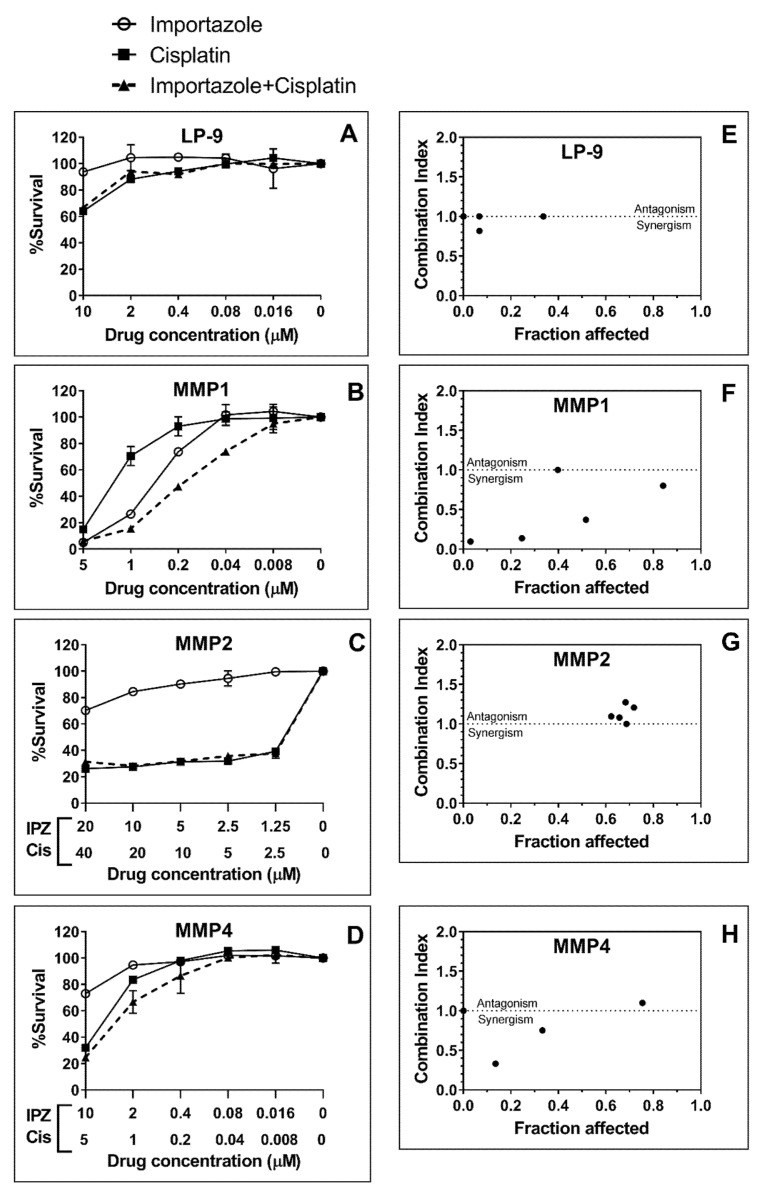
Cytotoxicity produced by IPZ and cisplatin (Cis) in LP-9 and primary cells MMP1, MMP2, and MMP4. (**A–D**) The dose-response cell survival was assessed after the cells were exposed to various concentrations of IPZ, Cis, or a combination of both (dashed lines) for 72 h of treatment. (**E–H**) The fraction affected-CI plots were constructed by CalcuSyn software, showing synergism (CI < 1), additive effect (CI = 1), and antagonism (CI > 1). For MMP1 cells (F), CI values of <1 occurred at a wide range of inhibition levels, indicating synergy produced by the combination of IPZ and Cis.

Then, the same assay was performed on a set of three primary cell lines deriving from MPM patients, MMP1, MMP2, and MMP4 (Figure 10). The calculated IC_50_ for these cell lines varied considerably; however, interestingly, cells from patient-MMP1 were showed to be sensitive with a calculated IC_50_ in the range of 1–0.2 µM. Cells from MMP2 and by MMP4 patients appeared far less sensitive. The commercially available non-malignant mesothelial cell line LP-9 was shown to be resistant to IPZ (Figure 10). In term of comparison, we employed cisplatin on the same cell lines, and we found that MMP1 cells were more sensitive to IPZ than cisplatin and that the combination of IPZ with cisplatin sensitized MMP1 cells to cisplatin (Figure 10). We employed the software CalcuSyn to quantify the effects (synergism/inhibition) of the combination of IPZ with cisplatin and expressed as combination index (CI). For MMP1, the CI values were all <0.8 in the dose range of 0.008–1 µM, indicating that combined IPZ and cisplatin produced synergistic cytotoxic effects (Figure 10F), with the exception of the dose 5 µM with a CI = 1. A similar result was also observed in MMP4 cells, but the combined drug effect was lower than that of patient MMP1. In fact, the synergistic effect (CI < 1) occurred in a narrow dose range of 0.4–2 µM for IPZ and 0.2–1 µM for cisplatin.

An overview of the results and assays performed is also provided in Supplementary Materials Table S1.

## 3. Discussion

In the present work, we studied MPM cell lines in order to shed light on the discrimination between mesothelial CDGs and PGs. The rationale is that genes whose over-expression is relevant for sustaining the malignant phenotype in tissues are more likely than PGs to be also found over-expressed in permanent cancer-derived cell cultures. Although this should not be taken as a strict rule, it could represent a criterion for restricting the list of *bona fide* CDGs. The chosen candidates were *ASS1*, *EIF4G1*, *GALNT7*, *GLUT1*, *IGF2BP3*, *ITGA4*, *RAN*, *SOD1*, and *THBS2*. In the considered panel of MPM cell lines, we could not detect an over-expressed status for *GLUT1*, *ITGA4*, and *SOD1*, and therefore we did not proceed further with their functional characterization. The remaining genes showed an increased expression both at the mRNA and protein level, and their depletion with siRNAs was evaluated for the effects produced on proliferation, colony formation, migration, and caspases activity. The rationale is that the silencing of a CDG is more likely to impact the phenotypes associated with the malignant behavior than the silencing of a PG. We acknowledge that the results could be biased by the choice of the assays. However, we carried out a basic set of tests to allow a fast screening of genes, bearing in mind that some CDGs could be neglected. 

As expected, not all the MPM cell lines responded similarly to gene depletion. The in vitro silencing of *ASS1*, *GALNT7*, *IGF2BP3*, and *THBS2* induced reductions in the proliferation, clonogenic ability, migration, or caspase activity of some, but not all, MPM cell lines. The variable effects observed in response to gene depletion could depend on several factors, including the different genetic background of the original donors, the different pathways triggered during tumorigenesis and cancer progression, and the genetic changes that occurred during in vitro culturing. Moreover, the different histological subtypes from which cell lines are derived could play an important role. However, this does not exclude a role for *ASS1*, *GALNT7*, *IGF2BP3*, and *THBS2* as *bona fide* CDGs, which should be evaluated on a larger panel of cell lines with alternative assays. For example, IGF2BP3 is known to have angiogenic activity in glioblastoma and giant cell tumor [21,22], and more refined assays investigating these aspects should be foreseen in future studies. Nevertheless, our results suggest that *EIF4G1* and *RAN* are the two most likely CDGs driving mesothelial carcinogenesis. 

EIF4G1 is the scaffolding protein of the eukaryotic initiation factor 4 F (eIF4F) complex needed for cap-dependent mRNA translation and protein synthesis. Increased expression of EIF4G1, described in several types of human cancers [23,24,25,26], promotes tumor cell proliferation through the modulation of the MNK1–eIF4G–eIF4E signaling pathway [27]. The silencing or the inhibition of EIF4G1 caused decreased cyclin D1 and Rb protein levels, cell cycle delay, reduced cell viability, proliferation, clonogenic activity, cancer spheroid formation, as well as increased sensitivity to chemotherapeutic drugs [26]. We also found an increase in caspases-3 and -7 activity, which is in agreement with the findings reported on the increased apoptosis in non-small-cell lung carcinoma cells, via a direct interaction with ubiquitin-specific protease 10 (USP10) [25]. Thus, EIF4G1 acts as an oncoprotein [28] and represents an interesting CDG for further investigation as a possible therapeutic target for MPM. These findings corroborate recent work carried out by Jaiswal et al. [29] on several types of cancers, including MPM. The authors showed that the overexpression of *EIF4G1* was associated with a bad prognosis, leading them to conclude that EIF4G1 could be a novel potential target for therapeutic interventions [29]. Based on mRNA expression data for *EIF4G1* from TCGA datasets, they analyzed the survival of patients with different cancer, including mesothelioma. Their analysis revealed that patients with high *EIF4G1* expression had lower median survival compared to the patients with low/medium expression (for mesothelioma, *p*  =  0.0043). Moreover, the marked overexpression of *EIF4G1* mRNA across human cancers agreed with findings from immunohistochemistry of an increase in EIF4G1 protein levels in cancer tissues derived from different organs as a result of amplification and/or mRNA up-regulation.

The results from the phenotypic assays also showed that *RAN* depletion impacted on proliferation, clonogenicity, and caspase activities of MPM cells but not MeT-5A cells. Hence, MPM cells seem to rely on RAN signaling for their survival and proliferation. Xia and colleagues were the first to show that RAN is differentially overexpressed in human cancer when compared with normal tissues [30]. Thereafter, an increasing number of publications began to report RAN overexpression in human tumors [31,32,33,34], highlighting this status as correlated with reduced patient survival and a more aggressive phenotype. Interestingly, despite the presence of RAN protein in non-tumorigenic Met-5A cells, its silencing in this cell line did not elicit significant changes in proliferation, caspase activation, and colony formation ability compared to the treatment with siCTRL. This result is in agreement with previous studies where RAN silencing, in various normal cell types, was well tolerated and did not cause mitotic defects or cell death. This suggests a “cancer-specific” utilization of RAN signaling for maintenance of cell viability and that RAN could represent a suitable anti-cancer target [30]. This hypothesis was reinforced by the observation that IPZ (an inhibitor of the nuclear transport by hampering the binding between RAN and importin-β) inhibited the proliferation of MPM cell lines, and this finding was also confirmed in a primary culture from an MPM patient. The data on primary cells suggested that the cells from MMP1 patient could be responsive to IPZ and that IPZ could also enhance the sensitivity of cancer cells to cisplatin administration. Because of the large variability among MPM patients, it is likely that only a share of MPM patients could benefit from the use of IPZ. As a matter of fact, cells from patients MMP2 and MMP4 were not particularly responsive to IPZ, despite an increased sensitivity to cisplatin when co-administered with IPZ observed for MMP4 cells.

In summary, this study is the first showing that *EIF4G1* and *RAN* behave as *bona fide* CDGs for mesothelium. We suggest that *EIF4G1* and *RAN* upregulation could be part of the mechanisms of pleural tumorigenesis, warranting further investigation of these genes. In agreement with the idea that *RAN* is a CDG for MPM, we found that IPZ elicits toxic effects in immortalized MPM cell lines as well as in the primary cancer cells from MPM patients. Moreover, IPZ increased the sensitivity of MMP1 and MMP4 cells to cisplatin. Thus, it is conceivable that, once available, more specific RAN inhibitors could be beneficial for MPM patients overexpressing this target.

## 4. Materials and Methods

### 4.1. Cell Lines

The MeT-5A cell line was purchased from ATCC (Manassas, VA, USA); Mero-14, Mero-25, and IST-Mes2 were kindly donated by the National Research Council (Istituto Tumori di Genova) of Genova, Italy; NCI-H28 were kindly donated by collaborators of the Pharmaceutical Department of University of Pisa, Italy. LP-9 cells were from Coriell Institute (Camden, NJ, USA), MMP1, MMP2, and MMP4 mesothelial cell lines were isolated from patients’ who underwent surgery at the Thoracic Surgery Unit (Siena, Italy) for decortication, without prior chemotherapy or radiotherapy [35]. All specimens were collected from patients diagnosed for pleural mesothelioma (MMP1, MMP4: epithelioid; MMP2: biphasic) with their written consent. The trial was approved by the Human Research Ethics Committee of the University of Siena, Regione Toscana, Italy - Area Vasta Sud Est (code CCMESOLUNG, n°1, 11/07/2016). The culturing conditions are reported in Supplementary B for brevity.

### 4.2. RNA Isolation, cDNA Synthesis, and Quantitative Real-Time PCR (RT-qPCR)

In order to evaluate the mRNA expression of each gene, we employed RT-qPCR. *GLUT1*, *IGF2BP3*, and *THBS2* were evaluated using specific TaqMan assay probes (Life Technologies, Carlsbad, CA, USA): hs00892681_m1 (*GLUT1*), hs00559907_g1 (*IGF2BP3*), and 00170248_m1 (*THBS2*). *ASS1, EIF4G1, GALNT7, ITGA4, RAN*, and *SOD1* were evaluated using primers. Following the MIQE guidelines [36], and as reported in our previous works [6,37], *HPRT1*, *RPLP0*, and *TBP* were used as reference because they were shown to be stable. The primer sequences and their respective Tm, the reaction mixture, and the thermal cycling conditions for cDNA synthesis and RT-qPCR are reported in Supplementary B.

### 4.3. Protein Extraction and Western Blot

Cells were collected 96 h after seeding, i.e., 72 h after siRNA transfection, washed twice with ice-cold PBS (Euroclone, Milan, Italy), and harvested by centrifugation at 9300× *g* for 1 min, at 4 °C. Cell pellets were processed for Western blots as reported in Supplementary B. The following primary antibodies were used: ASS1 mouse monoclonal (dilution 1:2000; NBP2-37-518, Novus Biologicals, Centennial, CO, USA); EIF4G1 rabbit polyclonal (dilution 1:750, NBP1-04964 Novus Biologicals), GALNT7 rabbit polyclonal (dilution 1:15,000; NBP1-32-491 Novus Biologicals); GLUT1 mouse monoclonal (dilution 1:1500; 66290-1-lg, Proteintech, Rosemont, IL, USA), IGF2BP3 rabbit polyclonal (dilution 1:2000; 14642-1-AP, Proteintech), ITGA4 mouse monoclonal (dilution 1:1000, NBP2-37503 Novus Biologicals), RAN rabbit polyclonal (dilution 1:1000; NB100-91945, Novus Biologicals), SOD1 rabbit monoclonal (dilution 1:4000; NB110-57590, Novus Biologicals), and THBS2 goat polyclonal (dilution 1:500; sc-7655, Santa Cruz Biotechnology, Dallas, TX, USA). β-Actin mouse monoclonal was purchased from Millipore, Burlington, MA, USA (dilution 1:5000, MAB1501). As secondary antibodies we employed HRP (horseradish peroxidase)-conjugated goat anti-rabbit IgG (sc-2004, Santa Cruz Biotechnology) and HRP-conjugated goat anti-mouse IgG (sc-2005, Santa Cruz Biotechnology).

### 4.4. Gene Silencing

Gene silencing and phenotypic assays were performed on six genes found up-regulated in at least two different MPM cell lines. Lyophilized silencing RNA oligonucleotides (siRNAs) were purchased from Qiagen (Hilden, Germany) or Invitrogen Corporation (Carlsbad, CA, USA) and resuspended in the provided buffer at a final stock concentration of 20 µM. In each experiment they were used at a final concentration of 20 nM for Mero-14 and 50 nM for all the other cell lines, employing HiPerfect (Qiagen) as transfection reagent. As negative control (siCTRL), the “AllStars Negative Control siRNA” (SI03650318, Qiagen) was used. The panel of six genes was composed by *ASS1*, *EIF4G1*, *GALNT7*, *IGF2BP3, RAN*, and *THBS2*. Their specific siRNAs were tagged as siASS1-1 (SI04434255, Qiagen), siEIF4G1-1 (SI04189346, Qiagen), siGALNT7-1 (SI00424508, Qiagen), siIGF2BP3-1 (SI04344256, Qiagen), siRAN-1 (SI04950519, Qiagen), and siTHBS2-1 (HSS110726, Invitrogen).

### 4.5. Sulphorhodamine (SRB) Assay

One to three thousand cells, depending upon the cell line employed, were seeded in 96-well plates. The following day, one plate was fixed (this was our day 0), and four plates were treated with siRNA and analyzed at 2-day intervals for a total of 8 days. Cell viability was evaluated by sulforhodamine B (SRB) assay (Sigma-Aldrich), as previously described [37]. Plates were then read at OD 540 nm, using a Bio-Rad Imark microplate reader (Bio-Rad, Hercules, CA, USA). For combination studies with importazole (Cayman Chemical, MI, USA) and cisplatin (AdipoGen ^®^ Life Sciences, CA, USA), primary cell lines were seeded 24 h before treatment with the drugs, alone and in combination, and then incubated for a further 72 h. Control cells were treated with the same amount of vehicle used to deliver the molecules. All the experiments were conducted in duplicate with at least four replicates for each experiment.

### 4.6. Caspase-3 and -7 Assay

The luminescence assay Caspase-Glo^®^ 3/7 (Promega, Madison, WI, USA) was performed to measure apoptosis in siRNA treated cells. Cells were plated in 6-well plates, at various concentrations, and after 24 h they were treated with the proper siRNA. After 72 h, cells were trypsinized and about 15 × 10^3^ were transferred in a 96-well white plate (Corning, Corning, NY, USA). Caspase-3/7-Glo reagent was added in a 1:1 ratio to each well, and the plate was incubated at room temperature, protected from light, for 1 h. Luminescence was determined with the FLUOstar^®^ Omega microplate reader (BMG LABTECH, Offenburg, Germany).

### 4.7. Colony Formation Assay

One to three thousand cells, depending upon the cell line employed, were seeded in 96-well plates and treated with the proper siRNA after 24 h. The following day, cells were trypsinized and transferred into 6-well plates. After 14 days of incubation, the growth medium was removed, and cells were fixed and stained in 10% ethanol solution containing 0.1% crystal violet (Sigma Aldrich) at room temperature for 1 h. Cells were finally washed twice with PBS (Euroclone). The images of the plates were then acquired and analyzed with Image J (NIH, Bethesda, MD, USA). The staining intensity of each plate was used as a proxy for the number of colonies.

### 4.8. Wound-Healing Assay

Cells were seeded in a 6-well plate at various densities and, after 24 h, treated with the relevant siRNA. The following day, when cells reached confluence, a linear scratch in the confluent cell monolayer was made with a sterile pipette tip. The time of scratching was considered as T = 0 h. Cells were washed twice with a serum-free medium and incubated in a full medium. Cells were photographed with an optical microscope at 10X magnification connected to a computer 24, 48, and 72 h after the scratch. The migration was then evaluated on the images and measured using ZEN software by Zeiss (Oberkochen, Germany).

### 4.9. Importazole IC_50_ Calculation and Treatment of the Cell Lines

After seeding in 96-well plates, MeT-5A and MPM cells were incubated for 24 h at 37 °C, 5% CO_2_ and then treated with drug, importazole, and DMSO as vehicle. Cells were treated with serial dilutions of compounds at doses ranging from 0.24 to 500 μM, and dose-dependent effects of the chemical agent were determined after 24 h. Viability was assessed by adding 3-(4,5-Dimethyl-2-thiazolyl)-2,5-diphenyl-2H-tetrazolium bromide (MTT) solution (5 mg/mL, solved in PBS) (Sigma Aldrich) in each well (incubation time: 3 h at 37 °C). Upon removal of culture medium, MTT crystals were dissolved in 50 µL of DMSO, and absorbance at 595 nm and 655 nm was measured using the Bio-Rad Imark microplate reader (Bio-Rad, Hercules, CA, USA). IC_50_ values were calculated from a log([drug]) versus normalized response curve fit using a four-parameter analytical method (GraphPad Prism 7 Software). Three independent experiments, each in triplicate, were performed.

### 4.10. Data Analysis

Each phenotypic assay was performed in three independent experiments, each in triplicate. Western blot was performed in duplicate and for quantitative analysis, the intensities of the bands were quantified using Image Lab™ Software (Bio-Rad). β-Actin was used as a loading control in order to normalize the levels of detected protein. For the analyses of protein expression after gene silencing, the intensities of the bands were compared to that obtained following the treatment with siCTRL. For the quantification of the relative expression in MPM cell lines, the signal was compared to that obtained in MeT-5A cells. RT-qPCRs were repeated three times, and the relative expression of each gene in MPM cells (compared to that in MeT-5A) was calculated, as suggested in MIQE guidelines [36]. The statistical analysis of the time-courses was performed with multifactor analysis of variance (MANOVA), whereas all the two-sample comparisons were evaluated with a two-tailed Student’s t-test, by using 0.05 as the nominal significance threshold. Data analysis and summary graphs were produced with GraphPad Prism 7 (San Diego, CA, USA). The cytotoxic interaction between importazole and cisplatin was analyzed by the software CalcuSyn 2.11 and expressed as combination index (CI). The CI value enabled the quantitative definition of additivity (CI = 1), synergism (CI < 1), or antagonism (CI > 1).

## Figures and Tables

**Table 1:** IC_50_ of importazole (IPZ) in Met-5A and MPM cells. All values are in μM. The calculated IC_50_ values, for each cell line, are reported with 95% confidence interval, and the computed *r^2^* for nonlinear regression.

	Importazole		
	IC_50_ (μM)	95%Cl	*r* ^2^
**MeT-5A**	20.23	13.1–36.05	0.963
**Mero-14**	10.92	7.67–15.33	0.935
**Mero-25**	24.06	20.61–27.78	0.973
**IST-Mes2**	19.34	15.36–23.82	0.962
**NCI-H28**	20.75	17.43–24.5	0.977
**MMP1**	1.86	0.55–6.43	0.962
**MMP2**	18.15	6.04–84.33	0.919
**MMP4**	12.30	1.40–90.75	0.912
**LP-9**	16	1.72–111.4	0.936

## Supplementary Materials

Supplementary materials can be found at https://www.mdpi.com/1422-0067/21/14/4856/s1.

## Abbreviations

CDG	Cancer driving gene
IPZ	Importazole
MPM	Malignant pleural mesothelioma
MTT	3-(4,5-Dimethyl-2-thiazolyl)-2,5-diphenyl-2H-tetrazolium bromide
PG	Passenger gene
SRB	Sulphorhodamine B
