# Supplementary material for: EIF4G1 and RAN as Possible Drivers for Malignant Pleural Mesothelioma
Source: Int J Mol Sci. 2020 Jul 9;21(14):4856. doi: 10.3390/ijms21144856 (PMC7402288; doi:10.3390/ijms21144856)
Supplement: Supplementary file 1 [file ijms-21-04856-s001.zip › ijms-820510 final supplementary/Appendix A.pdf]

# *EIF4G1* and *RAN* as possible drivers for Malignant Pleural Mesothelioma

**Irene Dell'Anno[1, ¥], Marcella Barbarino[2,3, ¥], Elisa Barone[1, ¥], Antonio Giordano[2,3], Luca Luzzi[4], Maria Bottaro[2], Loredana Migliore[1], Silvia Agostini[1], Alessandra Melani[1], Ombretta Melaiu[1,5], Calogerina Catalano[1,6], Monica Cipollini[1], Roberto Silvestri[1], Alda Corrado[1,7], Federica Gemignani[1, §], Stefano Landi[1, §, *]**

1 Department of Biology, Genetic Unit, University of Pisa, Pisa, Italy; irene.dellanno@biologia.unipi.it (I.D.); elisa_barone@ymail.com (E.B.); loredana.migliore@student.unisi.it (L.M.); silvietta.agostini@gmail.com (S.A.); alessandra-29@hotmail.it (A.M.); ombretta.melaiu@unipi.it (O.M.); cal.catalano@gmail.com (C.C.); monica.cipollini@unipi.it (M.C.); r.silvestri17@gmail.com (R.S.); corradoalda@gmail.com (A.C.); federica.gemignani@unipi.it (F.G.).

2 Department of Medical Biotechnologies, University of Siena, Siena, Italy; marcella.barbarino@unisi.it (M.B.[¥]); president@shro.org (A.G.); mariaeusebia.bottaro@gmail.com (M.B.).

3 Sbarro Institute for Cancer Research and Molecular Medicine, Center for Biotechnology, College of Science and Technology, Temple University, Philadelphia, Pennsylvania.

4 Department of Medicine, Surgery and Neurosciences, Siena University Hospital, Siena, Italy; dr.luca.luzzi@gmail.com (L.L.).

5 Immuno-Oncology Laboratory, Department of Paediatric Haematology/Oncology and of Cell and Gene Therapy, Ospedale Pediatrico Bambino Gesù, IRCCS, Rome, Italy.

6 Department of Internal Medicine V, University of Heidelberg, Heidelberg, Germany.

7 Deprtment of Bioscience, University of Milan, Milan, Italy.

* Correspondence: Stefano Landi, stefano.landi@unipi.it

¥ The authors contributed equally to this work.

§ The authors contributed equally to this work

## SUPPLEMENTARY RESULTS

### *ASS1* Screening.

Western blots showed that ASS1 had a positive staining, at various extents, in all the cell lines [Fig. S4]. A low expression was observed in MeT-5A. MPM cells showed varied expressions: low (i.e. a relative expression ≤1) in Mero-14 and NCI-H28, high (i.e. a relative expression ≥1) in Mero-25 and IST-Mes2 cells with relative intensities of 3-fold (P=0.044) and 2-fold (P=0.049), respectively. A similar trend was observed also for *ASS1* mRNA, with a low relative expression in Mero-14 and NCI-H28 (P<0.001 for both cells) and a high relative expression in Mero-25 and IST-Mes2 cells (1.50-fold, P<0.001 and 2.3-fold, P<0.001, respectively) [Fig. S4]. Thus, the phenotypic assays following *ASS1* silencing were performed in Mero-25, IST-Mes2, and MeT-5A cell lines. The siRNA targeting *ASS1*, now on called siASS1-1, was effective at various extents, leading to a residual expression of the mRNA ranging from 0.10X (MeT-5A) to 0.15X (IST-Mes2) as compared to siCTRL [Fig. S5]. The silencing efficiency was measured also at protein level: ASS1 depletion was efficient, at various extents, in all cell lines under examination, with a residual expression ranging from 0.40X (IST-Mes2) to 0.5X (Mero-25) [Fig. S5]. The depletion of *ASS1* significantly affected the proliferation of Mero-25 and IST-Mes2 cell lines [Fig. S6]. In Mero-25, proliferation decreased of about 40% at day 6 (P<0.001) and of 35% at day 8 after transfection (P<0.001). In IST-Mes2 the proliferation was reduced of about 23% at day 6 (P<0.001) and at day 8 (P<0.001). In MeT-5A, we did not observe any statistically significant difference following the transfection with siCTRL or siASS1-1 [Fig. S6]. Moreover, Mero-25 and IST-Mes2 cell lines showed a decreased ability in colony formation [Fig. S7] with an average decrease in the number of colonies of about 25% (P=0.0071) and 30% (P=0.0061), respectively. No effects were seen in MeT-5A cell line. *ASS1* silencing induced a slight increase of caspases activities, although not statistically significant, in the order of 1.3- and 1.2- fold for Mero-25 (P=0.21) and IST-Mes2 (P=0.42), respectively [Fig. S8]. Met-5A did not show

any changes after *ASS1* silencing, in comparison to siCTRL treatment [Fig. S8]. Finally, we did not observe any relevant and significant difference in migration ability after *ASS1* silencing in any of the cell lines under treatment [data not shown for brevity].

***EIF4G1* Screening.**

All the analysed cell lines showed a positive staining of EIF4G1 in the western blots. The relative expression of Mero-14 and IST-Mes2 was higher, close to the statistical significance, to the reference (respectively: 1.4-fold, P=0.08 and 1.7-fold, P=0.06) whereas the other MPM cell lines showed similar levels [Fig. 1A-B]. The results of RT-qPCR showed that MeT-5A, Mero-25, and NCI-H28 had similar levels of expression [Fig. 1C], whereas IST-Mes2 and Mero-14 had the highest expression (about 2-fold for both, compared to MeT-5A, P<0.001 and P=0.0045, respectively). Thus, MeT-5A, Mero-14, and IST-Mes2 were further evaluated following *EIF4G1* gene silencing. The siRNA, now on named siEIF4G1-1, was effective both at mRNA and protein level in all cell lines [Fig. 1]. The residual expression of *EIF4G1* mRNA ranged from 0.10X (IST-Mes2, P<0.001) to 0.30X (Mero-14 and MeT-5A, P<0.001 for both) as compared to siCTRL. At protein level, the residual expression ranged from <0.1X (MeT-5A, P=0.0033) to 0.46X (Mero-14, P=0.055) [Fig. 1D-E]. Compared to siCTRL, the treatment with siEIF4G1-1 induced a reduction (about -75%) of the proliferation rate in IST-Mes2 cells (P<0.001) [Fig. 2], that was not observed in MeT-5A or in Mero-14 cells. Overall, clonogenic assay showed a decreased colony formation ability, fourteen days after *EIF4G1* silencing, in all malignant cell lines but not in MeT-5A cells [Fig. 3]. The number of colonies were reduced of about 18% in Mero-14 (P=0.0088) and of 32% in IST-Mes2 (P=0.022) cells, as compared to siCTRL. MeT-5A cell line did not show any effect following siEIF4G1-1 treatment. *EIF4G1* depletion caused an increase in caspases 3 and 7 activity in all the cell lines, ranging from about 1.4X (IST-Mes2) to 1.6X (Mero-14), statistically significant at the level of 0.05

(with the exception of IST-Mes2 cells) when compared to siCTRL [Fig. 4]. The wound healing assay showed no effects [data not reported for brevity].

***GALNT7* Screening.**

The expression of GALNT7 was highly variable also within the same cell lines and the replicates of the western blots showed variable intensities. On the average, an abundant relative expression of GALNT7 was observed for Mero-14 and IST-Mes2 (about 6.5X for both, compared to MeT-5A) [Fig. S9]. The mRNA analysis showed the same pattern of expression [Fig. S9]. We used siGALNT7-1 in MeT-5A, Mero-14, and IST-Mes2 cells siGALNT7-1 acted efficiently, since *GALNT7* mRNA and protein levels were strongly reduced in all cell lines, with a residual expression ranging from 0.10X (IST-Mes2, $P<0.001$) to 0.30X (Mero-14, $P<0.001$) for mRNA and from about 0.10X (IST-Mes2, $P=0.013$) to 0.20X (MeT-5A, $P<0.05$) for the protein [Fig. S10]. *GALNT7* silencing slightly impaired the proliferation of MPM cell lines [Fig.S11]. We observed a decrease of about 30% in Mero-14 ($P=0.085$) and 20% in IST-Mes2 ($P=0.0035$), eight days after siGALNT7-1 treatment, whereas no significant effects were recorded in MeT-5A [Fig. S11]. *GALNT7* did not affect the clonogenic ability of MeT-5A and Mero-14 cells, whereas a reduction was shown in IST-Mes2 cells with a decrease of about 40% in colonies number ($P<0.05$, Fig. S12). No significant changes were induced by siGALNT7-1 in the caspases' activity: we only observed, approximately, a slight decrease, up to 30%, statistically not-significant, in MeT-5A and in Mero-14, compared to siCTRL [data not shown for brevity]. We did not record any significant effect on the migration abilities of the tested cells after *GALNT7* silencing [data not shown for brevity].

***IGF2BP3* Screening.**

Two MPM cell lines, Mero-14 and IST-Mes2, showed a statistically significant up-regulated relative expression of IGF2BP3 protein (Mero-14, $P=0.0093$; IST-Mes2, $P=0.039$) [Fig. S13]. These findings were also confirmed at mRNA level, with high levels of *IGF2BP3* in Mero-14 ($P=0.026$) and in IST-

Mes2 (P=0.0052) versus MeT-5A [Fig. S13]. Thus, gene silencing was carried out in MeT-5A, Mero-14, and IST-Mes2. The siRNA, now on called siIGF2BP3-1, was effective at mRNA level for all the cell lines, at various extents, ranging from 0.12X (Mero-14, P<0.001) to 0.60X (MeT-5A, P=0.0025) as compared to the siCTRL [Fig. S14]. The western blots showed the same pattern, with a residual expression ranging from 0.5X (IST-Mes2, P=0.0013) to 0.7X (MeT-5A, P=0.07) [Fig. S14]. In spite the low reduction of IGF2BP3 protein after gene silencing in MeT-5A (likely because the low basal expression), we proceed anyway with the phenotypic study in the three cell lines. *IGF2BP3* silencing reduced the growth of Mero-14 cells, starting from day 4, reaching a maximum reduction of 54.58% at day 8 (P<0.001). In MeT-5A and IST-Mes2, the proliferation was not affected significantly compared to siCTRL treatment [Fig. S15]. The clonogenic ability was not affected by the treatment with siIGF2BP3-1 [data not shown for brevity], whereas caspase -3 and -7 activities showed an increase of about 20-40% in the three cell lines, not statistically significant [Fig. S16]. Similarly, lack of effects was recorded in the wound healing assay for MeT-5A and IST-Mes2. Mero-14 cells showed a decreased migration ability following siIGF2BP3-1 treatment, of about 25% (P=0.035) [Fig. S17].

***RAN* Screening.**

The protein RAN showed an elevated relative expression in all MPM cell lines [Fig. 5]. The measured intensities were 1.5-fold in Mero-14 (P=0.13), 1.75-fold in Mero-25 (P=0.011), 1.70-fold in IST-Mes2 (P=0.068) and 2.2-fold in NCI-H28 (P=0.024). The mRNA analysis confirmed this pattern for Mero-14 (P<0.001), Mero-25 (P=0.048) and IST-Mes2 (P<0.001), but not for NCI-H28, where mRNA basal levels of *RAN* resulted downregulated of 0.40-fold versus MeT-5A (P<0.001) [Fig. 5A-C]. However, we silenced *RAN* in MeT-5A and all the MPM cells. The siRNA, now on called siRAN-1, worked efficiently, at various extents, in four cell lines with a residual expression, of the mRNA, ranging from 0.1X (Mero-14, P<0.001) to 0.50X (NCI-H28, P=0.031) as compared to siCTRL

[Fig. 5F]. At protein level the residual expression ranged from 0.20X (IST-Mes2, P=0.002) to 0.90X (Mero-25, not significant) as compared to that measured following siCTRL treatment [Fig. 5D-E]. Cellular growth of all MPM cell lines was clearly affected after *RAN* silencing and this impairment was appreciable starting from four days after the treatment. At day 8, as compared to siCTRL, the proliferation rate dropped by an average of 19.3% NCI-H28 (P=0.012), of 50% in Mero-14 and IST-Mes2 (P<0.001, for both cell lines), and of 71.6% In Mero-25 (P<0.001). MeT-5A showed only a slight reduction, statistically not significant [Fig. 6]. As illustrated in Fig. 7, *RAN* depletion strongly impaired the capability of colony formation of all MPM cell lines, with Mero-14 being the most affected (-98%, P=0.0065), but not of MeT-5A [Fig. 7]. Gene silencing caused an increase of the activity of caspases 3 and 7 in all MPM cell lines, ranging from 1.2-fold (Mero-14, P<0.001) to about 2-fold (IST-Mes2, P=0.032) [Fig. 8]. Lack of effects was recorded for MeT-5A cells [Fig. 8]. The migration capacity of MPM cells was unaffected, whereas MeT-5A was the only cell line to show a reduced activity after gene silencing (-65%, P=0.086) [Fig. S18].

***THBS2* Screening**

The protein THBS2 showed to be expressed at various extents in three MPM cell lines: Mero-14, Mero-25, and, weekly, in IST-Mes2 [Fig. S19]. The relative intensities were about 3.2-fold (P<0.05), 2.2-fold (P=0.08) and 1.3-fold (P=0.092) higher than that of MeT-5A, respectively. The mRNA analysis showed a weak expression in MeT-5A and in NCI-H28 and confirmed a high relative expression in Mero-14 (48.5-fold, P=0.0036), Mero-25 (4.6-fold, P=0.11), and IST-Mes2 (11-fold, P<0.001) [Fig. S19]. Thus, *THBS2* was silenced in MeT-5A and in Mero-14, Mero-25, and IST-Mes2. The siRNA, now on called siTHBS2-1, worked at various extents, with a residual mRNA expression ranging from 0.1X (Mero-14, P<0.001) to 0.58X (MeT-5A, P=0.047) as compared to siCTRL [Fig. S20]. However, at protein level, the depletion of THBS2 occurred only in Mero-14 and IST-Mes2 and we did not proceed further with the phenotypic screening on Mero-25 [Fig. S20]. The

proliferation rate was affected only in IST-Mes2, and 8 days following siTHBS2 treatment we recorded a statistically significant decrease of about 63.6% (P=0.044) versus siCTRL treatment [Fig. S21]. Mero-14 and MeT-5A did not show any significant difference. The clonogenic ability was clearly impaired [Fig. S22]. Following *THBS2* silencing, we detected a significant drop in colony number of 70% (P=0.002). No changes in clonogenic ability of non-malignant Met5A and Mero-14 cell lines were observed [Fig. S22]. The depletion of *THBS2* induced an increase of caspase 3/7 activity in MeT-5A and Mero-14 of about 15% and 30% respectively, compared to the control (not statistically significant) [Fig. S23]. In IST-Mes2 cells we did not observe any significant change in caspases activities. The wound healing assay did not show significant differences between cells treated with siCTRL or siTHBS2-1 [Data not shown for brevity].
