# Supplementary material for: EIF4G1 and RAN as Possible Drivers for Malignant Pleural Mesothelioma
Source: Int J Mol Sci. 2020 Jul 9;21(14):4856. doi: 10.3390/ijms21144856 (PMC7402288; doi:10.3390/ijms21144856)
Supplement: Supplementary file 1 [file ijms-21-04856-s001.zip › ijms-820510 final supplementary/Appendix B.pdf]

EIF4G1 and *RAN* as possible drivers for Malignant Pleural Mesothelioma

Irene Dell'Anno[1, ¥], Marcella Barbarino[2,3, ¥], Elisa Barone[1, ¥], Antonio Giordano[2,3], Luca Luzzi[4], Maria Bottaro[2], Loredana Migliore[1], Silvia Agostini[1], Alessandra Melani[1], Ombretta Melaiu[1,5], Calogerina Catalano[1,6], Monica Cipollini[1], Roberto Silvestri[1], Alda Corrado[1,7], Federica Gemignani[1, §], Stefano Landi[1, §, *]

1 Department of Biology, Genetic Unit, University of Pisa, Pisa, Italy; irene.dellanno@biologia.unipi.it (I.D.); elisa_barone@ymail.com (E.B.); loredana.migliore@student.unisi.it (L.M.); silvietta.agostini@gmail.com (S.A.); alessandra-29@hotmail.it (A.M.); ombretta.melaiu@unipi.it (O.M.); cal.catalano@gmail.com (C.C.); monica.cipollini@unipi.it (M.C.); r.silvestri17@gmail.com (R.S.); corradoalda@gmail.com (A.C.); federica.gemignani@unipi.it (F.G.).
2 Department of Medical Biotechnologies, University of Siena, Siena, Italy; marcella.barbarino@unisi.it (M.B.¥); president@shro.org (A.G.); mariaeusebia.bottaro@gmail.com (M.B.).
3 Sbarro Institute for Cancer Research and Molecular Medicine, Center for Biotechnology, College of Science and Technology, Temple University, Philadelphia, Pennsylvania.
4 Department of Medicine, Surgery and Neurosciences, Siena University Hospital, Siena, Italy; dr.luca.luzzi@gmail.com (L.L.).
5 Immuno-Oncology Laboratory, Department of Paediatric Haematology/Oncology and of Cell and Gene Therapy, Ospedale Pediatrico Bambino Gesù, IRCCS, Rome, Italy.
6 Department of Internal Medicine V, University of Heidelberg, Heidelberg, Germany.
7 Deprtment of Bioscience, University of Milan, Milan, Italy.
* Correspondence: Stefano Landi, stefano.landi@unipi.it
¥ The authors contributed equally to this work.
§ The authors contributed equally to this work

SUPPLEMENTARY MATERIALS AND METHODS

Cell lines.

Culturing conditions: MeT-5A are grown in Medium 199 with HEPES (Life Technologies), supplemented with 3.3 nM Epidermal Growth Factor (EGF) (Life Technologies), 870 nM Insulin (Life Technologies) and 400 nM Hydrocortisone (Sigma Aldrich). Mero-14, Mero-25 and IST-Mes2 are grown in DMEM medium (Euroclone) and NCI-H28 in RPMI medium (Euroclone). All media were supplemented with 10% FBS and 1% Penicillin Streptomycin (all purchased from Euroclone). LP-9, MMP1, MMP2 and MMP4 cell lines are cultured in Medium 199 (Euroclone), supplemented with 2 mmol/L l-glutamine (Euroclone), 100 U/mL penicillin, 100 µg/mL streptomycin (Euroclone), 10% FBS (Euroclone), 20 ng/mL hEGF (Sigma-Aldrich), 0.4 µg/ml hydrocortisone (Sigma-Aldrich). All cell lines were routinely passaged every 1–2 weeks. Cells were grown at 37°C and 5% CO_2.

RNA isolation, cDNA synthesis and Quantitative Real-Time PCR (RT-qPCR)

Cells were harvested 72 hours after siRNA transfection and washed in PBS. Total RNA was isolated using RNeasy Mini Kit (Qiagen) according to the manufacturer instructions. Concentration of clean-up RNA was determined using the Qubit® Fluorometer with the Qubit® RNA HS Assay Kit (Thermo Scientific). The reverse transcription (RT) was performed with iSCRIPT cDNA Synthesis Kit (Bio-Rad) using 1 µg of total RNA in a final reaction volume of 20 µL. The thermal cycling conditions were 5 min at 25 °C, 30 min at 42 °C and 5 min at 85 °C. To evaluate mRNA expression of each gene, before and after siRNA depletion, we used RT-qPCR. We used three reference genes, as *HPRT1, RPLP0* and *TBP*. *ASS1, EIF4G1, GALNT7, ITGA4, RAN* and *SOD1* were evaluated using primers. The reaction mixture consisted in 2 µL of cDNA template, 1 µL of each primer (Reverse/Forward, 10 µM), 5 µL HOT FIREPol® EvaGreen® qPCR Mix Plus (ROX) (Solis BioDyne) and 7 µL of deionized H_2O. The thermal cycling conditions were 15 min at 95 °C followed by 40 cycles at 95 °C for 15 s and Tm°C for 30 s. *GLUT1*, *IGF2BP3* and *THBS2* were evaluated using specific

TaqMan Assay probes (Life Technologies): hs00892681_m1 (*GLUT1*), hs00559907_g1 (*IGF2BP3*) and 00170248_m1 (*THBS2*). TaqMan Assay probes for reference genes were hs99999902_m1 (RPLP0), hs00427620_m1 (*TBP*) and hs01003267_m1 (*HPRT1*) (Life Technologies). The reaction mixture for TaqMan Assay consisted in: 10 µL 5X HOT FIREPol® Probe qPCR Mix Plus (no ROX) (Solis BioDyne), 7 µL of deionized H_2O, 2 µL of cDNA template and 1 µL of specific TaqMan Assay probe. The thermal cycling conditions were 15 min at 95 °C followed by 40 cycles at 95°C for 15 s and 60°C for 1 min.

Protein Extraction and Western Blot.

For protein extraction and western blots, cell pellets were suspended in ICE-cold RIPA buffer, containing PMSF, protease and phosphatase inhibitor (all purchased from Sigma Aldrich). After 15 min of ice-incubation the extracts were clarified by centrifugation at 16000 g for 15 min, at 4 °C. The protein concentration was determined with BCA Total protein colorimetric assay QuantumProtein (Euroclone). Lysates were incubated with 4x Laemmli Sample Buffer (Bio-Rad), according to the manufacturer instructions, for 10 min at 99 °C. An amount of 10 µg of proteins for each sample was loaded onto 10-15% SDS polyacrylamide gel. Proteins were transferred onto a nitrocellulose membrane (Bio-Rad). Running Buffer was purchased from Bio-Rad. The membrane was blocked for 1 h with 5% w/v non-fat dry milk (Bio-Rad) in TBS buffer (Thermo Scientific) containing 0.05% Tween 20 (Sigma Aldrich), washed, and successively incubated with different primary antibodies at 4 °C overnight. The membranes were washed three times for 8 min and incubated with the HRP-conjugated secondary antibody for 1 h at room temperature. After washing, three times for 8 min, the blot was exposed to Immobilon ™ Western (Millipore) and the image was acquired with Chemidoc MP (Bio-Rad). The intensity of the bands was quantified using Image Lab™ Software (Bio-Rad).

Primers employed for RT-qPCR assays.

Gene	Primer Sequences (Forward)	Primer Sequences (Reverse)	Tm (°C)
ASS1	ATTGACATCGTGGAGAACCG	GCCTCGATGTCTAAATGAGCA	60°C
EIF4G1	CTGTGTGACGAGCAGAAGGA	CCCAACTGTAGAAGGCATCC	59°C
GALNT7	AGTGGTCCTCTGGTCTTCCC	GCATGGGGTCATTGACATCT	60°C
HPRT1	GATGGTCAAGGTCGCAAG	GGGCATATCCTACAACAAACTT	62°C
ITGA4	AGATGCAGGATCGGAAAGAA	CCCCAACCACTGATTGTCTC	60°C
RAN	CAGGAGAAATTCGGTGGACT	ATGCCAGTTAGGCACATTCTT	60°C
RPLP0	CCTCATATCCGGGGGAATGTG	GCAGCAGCTGGCACCTTATTG	60°C
SOD1	AGGCCCCTTAACTCATCT	CTACAGGTACTTTAAAGCAACTCT	60°C
TBP	GCGGTTTGCTGCGGTAATC	TCTGGACTGTTCTTCACTCTTGG	57°C
