# Supplementary material for: EIF4G1 and RAN as Possible Drivers for Malignant Pleural Mesothelioma
Source: Int J Mol Sci. 2020 Jul 9;21(14):4856. doi: 10.3390/ijms21144856 (PMC7402288; doi:10.3390/ijms21144856)
Supplement: Supplementary file 1 [file ijms-21-04856-s001.zip › ijms-820510 final supplementary/ijms-820510 Supplementary Figuress.pdf]

EIF4G1 and *RAN* as possible drivers for Malignant Pleural Mesothelioma

Irene Dell'Anno[1, ¥], Marcella Barbarino[2,3, ¥], Elisa Barone[1, ¥], Antonio Giordano[2,3], Luca Luzzi[4], Maria Bottaro[2], Loredana Migliore[1], Silvia Agostini[1], Alessandra Melani[1], Ombretta Melaiu[1,5], Calogerina Catalano[1,6], Monica Cipollini[1], Roberto Silvestri[1], Alda Corrado[1,7], Federica Gemignani[1, §], Stefano Landi[1, §, *]

[1] Department of Biology, Genetic Unit, University of Pisa, Pisa, Italy; irene.dellanno@biologia.unipi.it (I.D.); elisa_barone@ymail.com (E.B.); loredana.migliore@student.unisi.it (L.M.); silvietta.agostini@gmail.com (S.A.); alessandra-29@hotmail.it (A.M.); ombretta.melaiu@unipi.it (O.M.); cal.catalano@gmail.com (C.C.); monica.cipollini@unipi.it (M.C.); r.silvestri17@gmail.com (R.S.); corradoalda@gmail.com (A.C.); federica.gemignani@unipi.it (F.G.).

[2] Department of Medical Biotechnologies, University of Siena, Siena, Italy; marcella.barbarino@unisi.it (M.B.[¥]); president@shro.org (A.G.); mariaeusebia.bottaro@gmail.com (M.B.).

[3] Sbarro Institute for Cancer Research and Molecular Medicine, Center for Biotechnology, College of Science and Technology, Temple University, Philadelphia, Pennsylvania.

[4] Department of Medicine, Surgery and Neurosciences, Siena University Hospital, Siena, Italy; dr.luca.luzzi@gmail.com (L.L.).

[5] Immuno-Oncology Laboratory, Department of Paediatric Haematology/Oncology and of Cell and Gene Therapy, Ospedale Pediatrico Bambino Gesù, IRCCS, Rome, Italy.

[6] Department of Internal Medicine V, University of Heidelberg, Heidelberg, Germany.

[7] Deprtment of Bioscience, University of Milan, Milan, Italy.

* Correspondence: Stefano Landi, stefano.landi@unipi.it

¥ The authors contributed equally to this work.

§ The authors contributed equally to this work

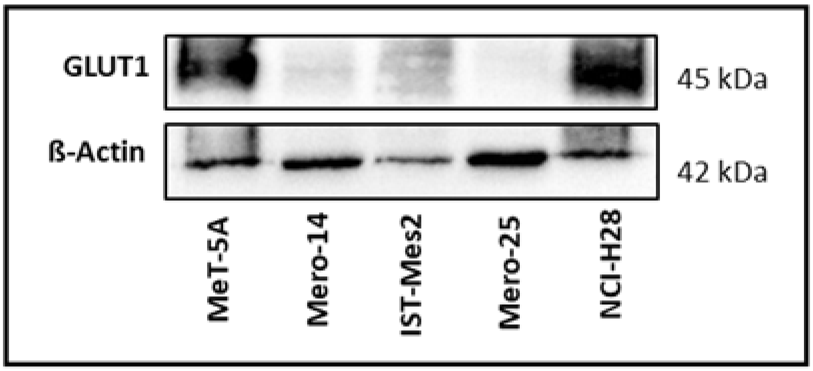

A

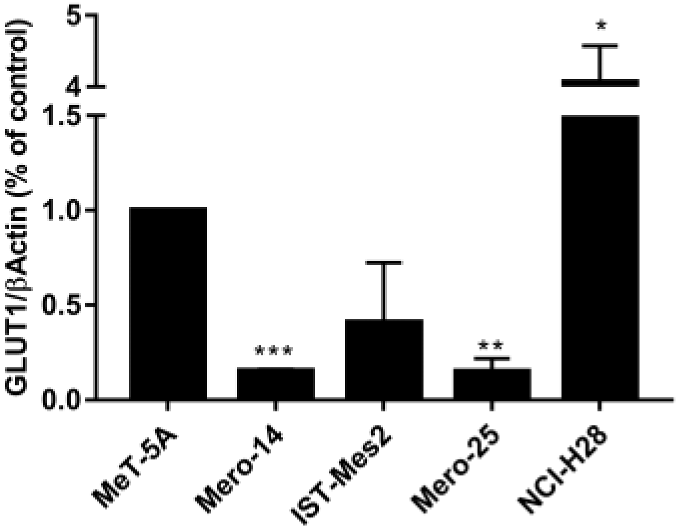

B

Fig. S1. Basal expression of GLUT1 in non-malignant MeT-5A and a panel of MPM cells, as Mero-14, Mero-25, IST-Mes2 and NCI-H28. A: Picture representing protein levels of GLUT1, in MeT-5A and MPM cell lines. ß-Actin was used as reference. The present picture is representative of one of two experiment performed. **B:** Histogram reporting protein levels of GLUT1 normalized to ß-Actin, in MeT-5A and MPM cell lines. The histogram was generated by quantifying blots from two independent experiments. The intensity of the bands was normalized to ß-Actin and compared to MeT-5A lane. Data are expressed as mean ± SEM. Statistical significance is indicated by asterisk (*), where *=P<0.05; **=P<0.01; ***=P<0.001, compared to control MeT-5A cell line.

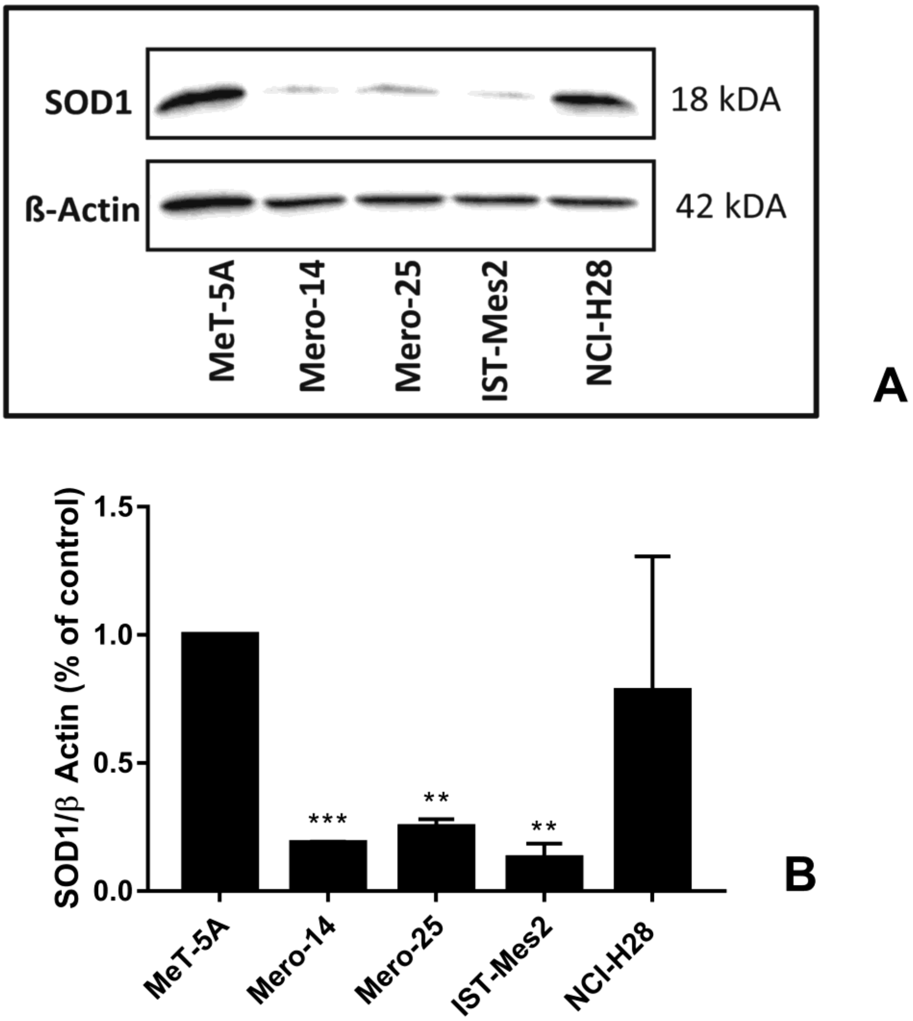

Fig. S2. Basal expression of SOD1 in non-malignant MeT-5A and a panel of MPM cells, as Mero-14, Mero-25, IST-Mes2 and NCI-H28. A: Picture representing protein levels of SOD1, in MeT-5A and MPM cell lines. ß-Actin was used as reference. The present picture is representative of one of two experiment performed. **B:** Histogram reporting protein levels of SOD1 normalized to ß-Actin, in MeT-5A and MPM cell lines. The histogram was generated by quantifying blots from two independent experiments. The intensity of the bands was normalized to ß-Actin and compared to MeT-5A lane. Data are expressed as mean ± SEM. Statistical significance is indicated by asterisk (*), where *=P<0.05; **=P<0.01; ***=P<0.001, compared to control MeT-5A cell line.

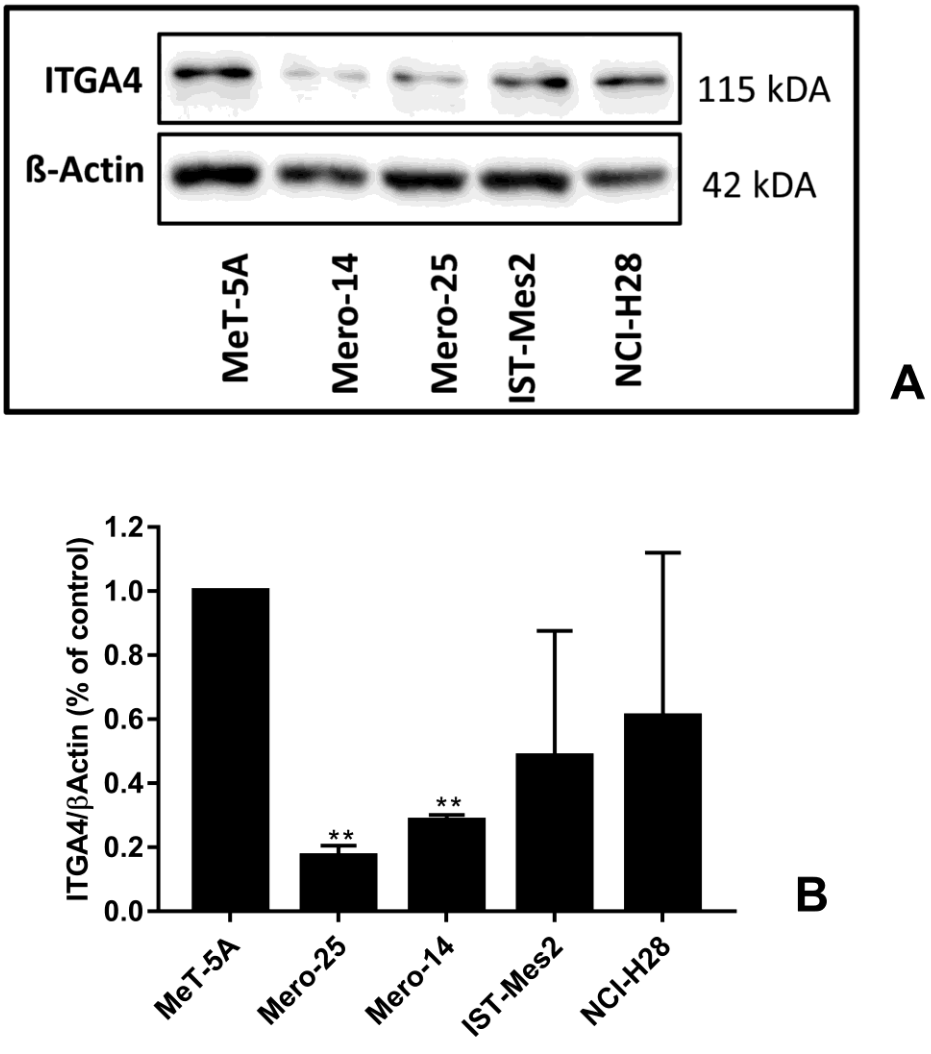

Fig. S3. Basal expression of ITGA4 in non-malignant MeT-5A and a panel of MPM cells, as Mero-14, Mero-25, IST-Mes2 and NCI-H28. A: Picture representing protein levels of ITGA4, in MeT-5A and MPM cell lines. ß-Actin was used as reference. The present picture is representative of one of two experiment performed. **B:** Histogram reporting protein levels of ITGA4 normalized to ß-Actin, in MeT-5A and MPM cell lines. The histogram was generated by quantifying blots from two independent experiments. The intensity of the bands was normalized to ß-Actin and compared to MeT-5A lane. Data are expressed as mean ± SEM. Statistical significance is indicated by asterisk (*), where *=P<0.05; **=P<0.01; ***=P<0.001, compared to control MeT-5A cell line.

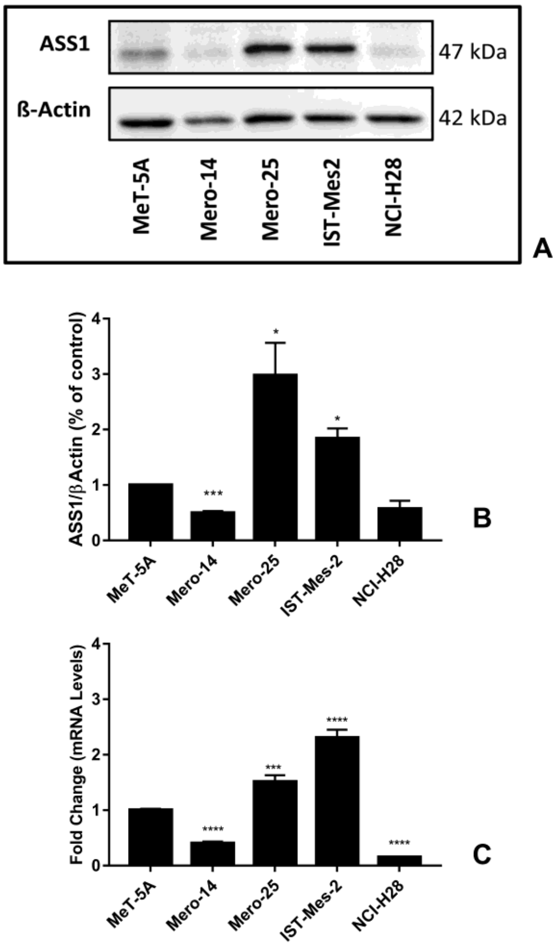

Fig. S4. Basal expression of ASS1 in non-malignant MeT-5A and a panel of MPM cells, as Mero-14, Mero-25, IST-Mes2 and NCI-H28. A: Picture representing protein levels of ASS1, in MeT-5A and mesothelioma cell lines. ß-Actin was used as reference. The present picture is representative of one of two experiment performed. **B:** Histogram reporting protein levels of ASS1, normalized to ß-Actin, in MeT-5A and MPM cell lines. The histogram was generated by quantifying blots from two independent experiments. The intensity of the bands was normalized to ß-Actin and compared to MeT-5A lane. Data are expressed as mean ± SEM. **C:** RT-qPCR showing fold changes of mRNA levels of *ASS1*, measured in MPM cell lines and related to MeT-5A, set to one. *RPLP0*, *HPRT1* and *TPB* were used for normalization. Error bars show the SEM from four independent experiments, each performed in triplicate. Statistical significance is indicated by asterisk (*), where *=P<0.05; **=P<0.01; ***=P<0.001, compared to control MeT-5A cell line.

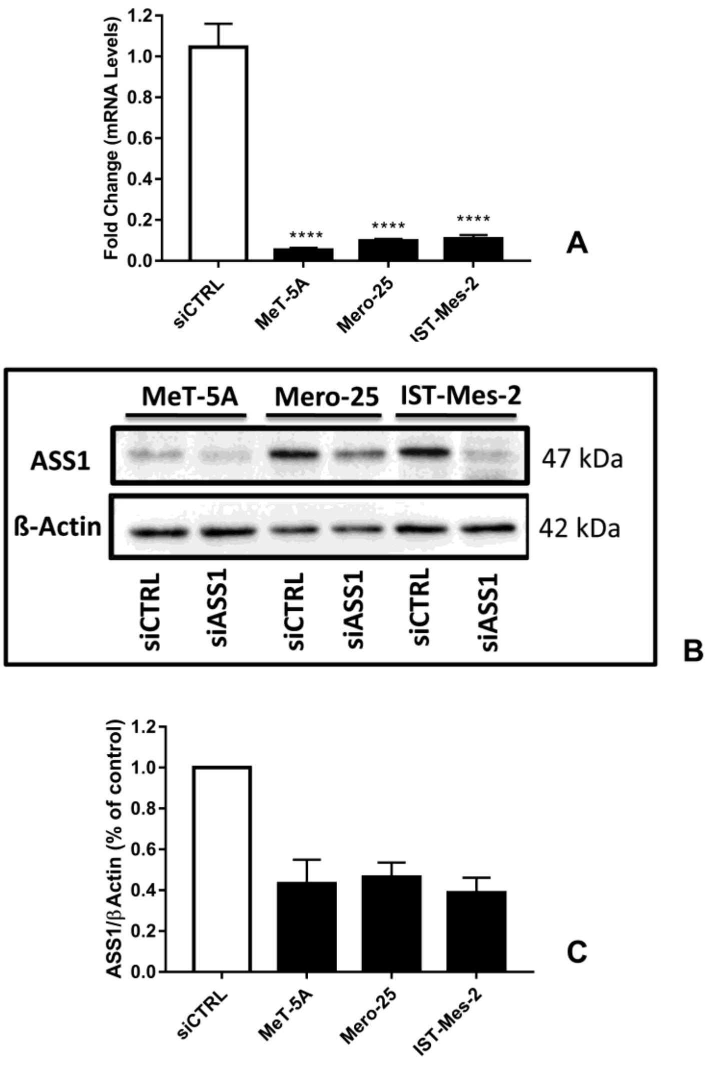

Fig. S5. Silencing of *ASS1* in mesothelial MeT-5A, Mero-25 and IST-Mes2 MPM cell lines, as Mero-25 and IST-Mes2. A: RT-qPCR showing, as fold change, the mRNA expression levels of *ASS1* in MeT-5A and MPM cells, after treatment with siASS1-1, related to their own siCTRL. *RPLP0*, *HPRT1* and *TPB* were used for normalization. Error bars are SEM, from three independent experiments, each performed in triplicate. **B:** Picture representing protein levels of ASS1, after its depletion through siASS1-1, in MeT-5A and MPM cells. ß-Actin was used as reference. The present picture is representative of one of two experiment performed. **C:** Histogram reporting protein levels of ASS1 normalized to ß-Actin, in MeT-5A and MPM cell lines. The histogram was generated by quantifying blots from two independent experiments. The intensity of the bands was normalized to ß-Actin and compared to siCTRL lane. Data are expressed as mean ± SEM. Statistical significance is indicated by asterisk (*), where *=P<0.05; **=P<0.01; ***=P<0.001, compared to control.

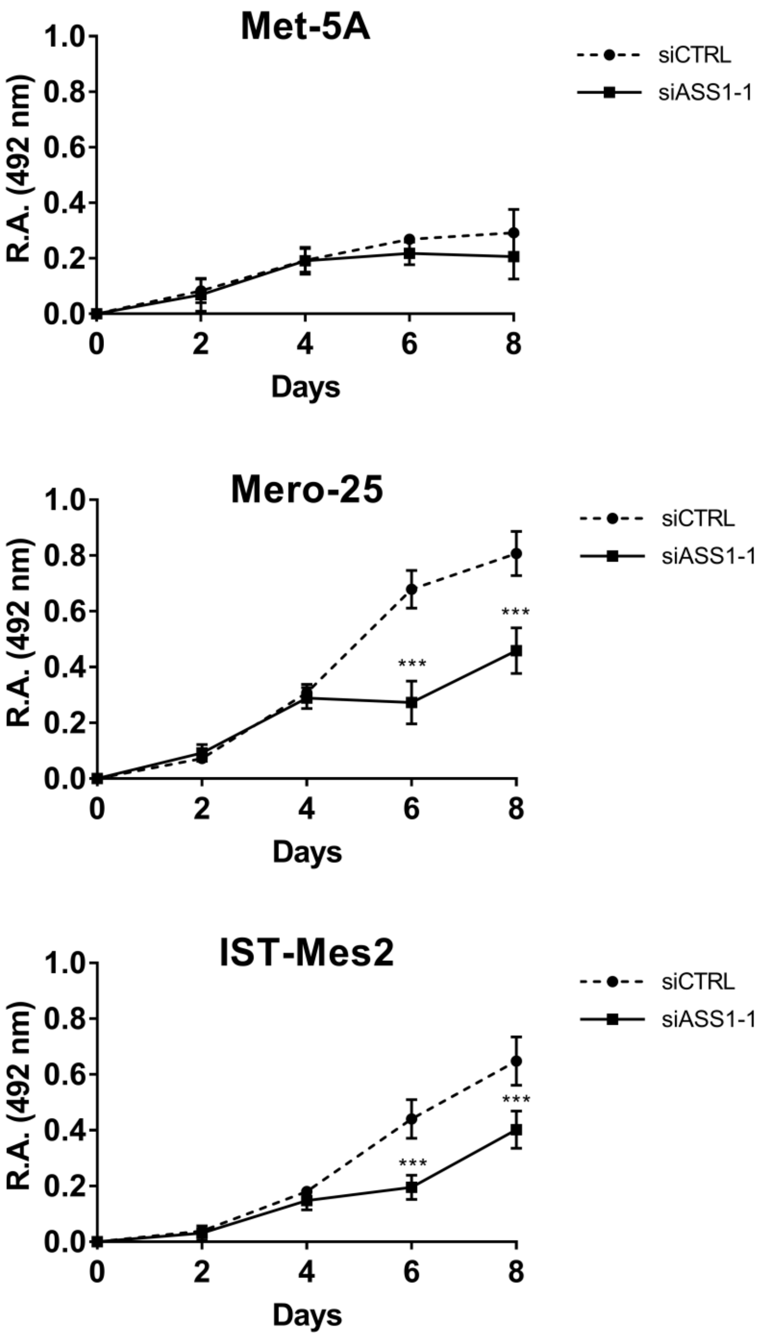

Fig. S6. ***ASS1*** **silencing and cellular growth**. Proliferation curves of MeT-5A and MPM cells, as Mero-25, and IST-Mes2. The acquisition of optical density, at 492 nm (Relative Absorbance, "R.A."), was made every two days after the day of transfection (that is Day 0) with siCTRL or siASS1-1. Error bars are SEM from three independent experiments, each performed in triplicate. Statistical significance is indicated by asterisk (*), where *=P<0.05; **=P<0.01; ***=P<0.001, compared to control treatment.

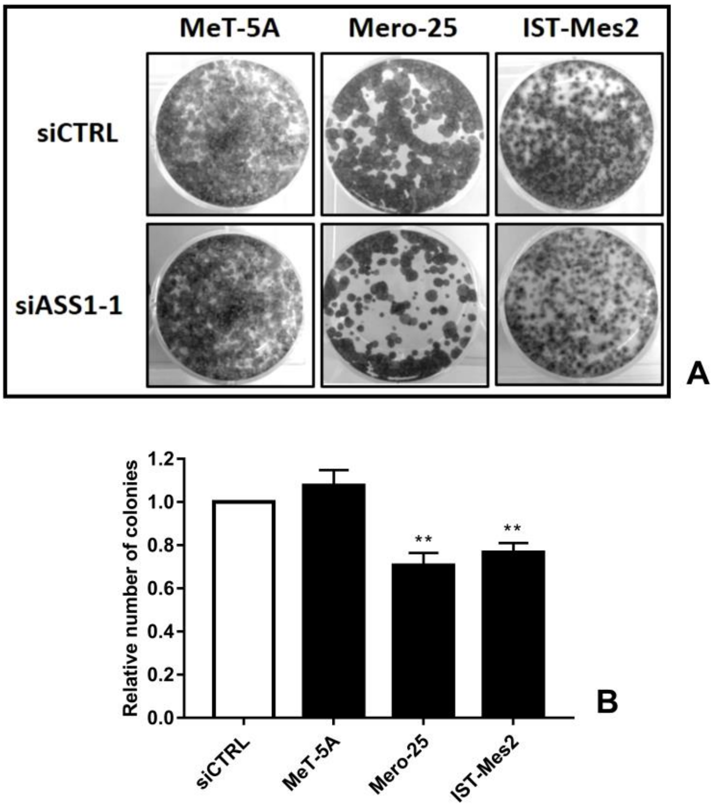

Fig. S7. *ASS1* silencing and colony formation ability. A: Colonies in MeT-5A and MPM cell lines fixed and stained 14 days after treatment with siCTRL or siASS1-1. This is a representative picture of one experiment. **B:** Histogram represents number of colonies measured 14 days after silencing with siASS1-1, compared to siCTRL. Error bars are SEM of three different experiments, each performed in triplicate. Statistical significance is indicated by asterisk (*), where *=P<0.05; **=P<0.01; ***=P<0.001, compared to control treatment.

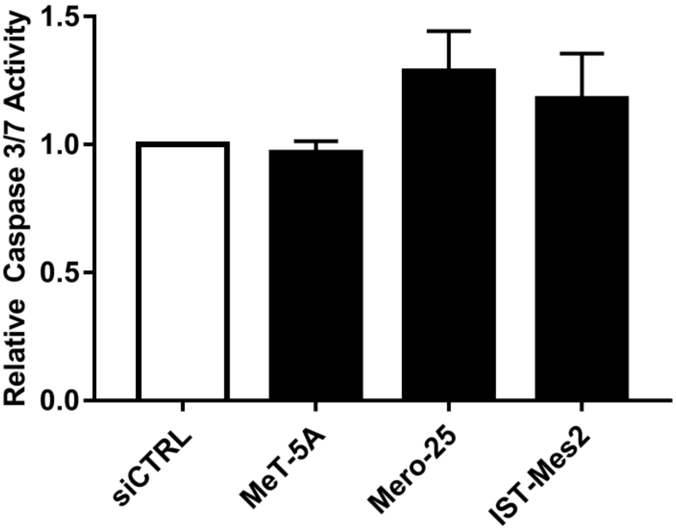

Fig. S8. ***ASS1*** **silencing and apoptosis.** Caspase activity measured in MeT-5A and MPM cells, following transfection with siCTRL (white bar) or siASS1-1. Error bars are the SEM of three independent experiments, each performed in triplicate. Statistical significance is indicated by *, where *=P<0.05; **=P<0.01; ***=P<0.001, compared to siCTRL treatment

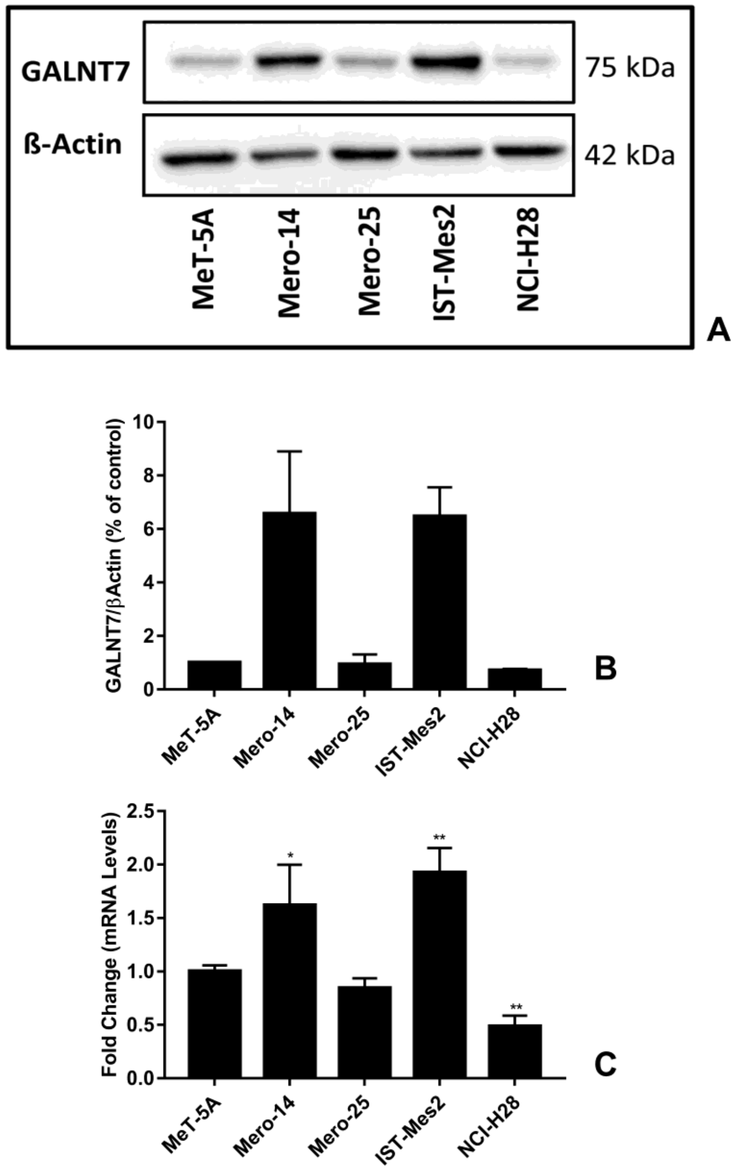

Fig. S9. Basal expression of GALNT7 in non-malignant MeT-5A and a panel of MPM cells, as Mero-14, Mero-25, IST-Mes2 and NCI-H28. A: Picture representing protein levels of GALNT7, in MeT-5A and mesothelioma cell lines. ß-Actin was used as reference. The present picture is representative of one of two experiment performed. **B:** Histogram reporting protein levels of EIF4G1, normalized to ß-Actin, in MeT-5A and MPM cell lines. The histogram was generated by quantifying blots from two independent experiments and normalizing the intensity of the bands to MeT-5A lane. Data are expressed as mean ± SEM. **C:** RT-qPCR showing fold changes of mRNA levels of *GALNT7*, measured in MPM cell lines and related to MeT-5A, set to one. *RPLP0*, *HPRT1* and *TPB* were used for normalization. Error bars show the SEM from three independent experiments, each performed in triplicate. Statistical significance is indicated by asterisk (*), where *=P<0.05; **=P<0.01; ***=P<0.001, compared to control MeT-5A cell line.

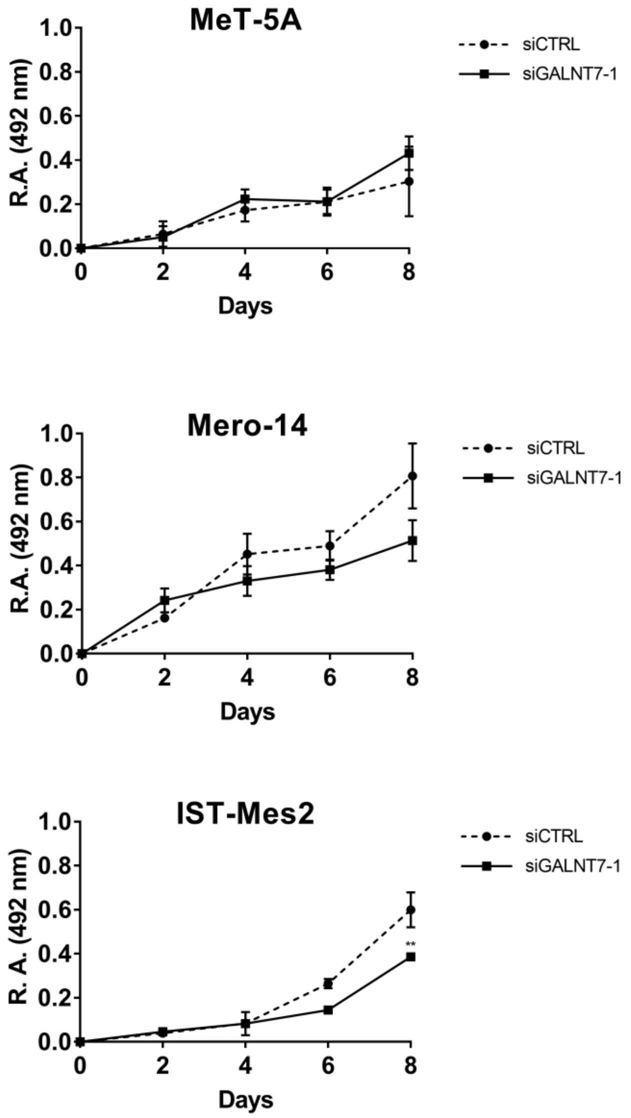

Fig. S10. Silencing of *GALNT7* in MeT-5A, Mero-14 and IST-Mes2 MPM cell lines. A: RT-qPCR showing, as fold change, the mRNA expression levels of *GALNT7* in MeT-5A and MPM cells, after treatment with siGALNT7-1, related to their own siCTRL. *RPLP0*, *HPRT1* and *TPB* were used for normalization. Error bars are SEM, from three independent experiments, each performed in triplicate. **B:** Picture representing protein levels of GALNT7, after its depletion through siGALNT7-1, in MeT-5A and MPM cells. ß-Actin was used as reference. The present picture is representative of one of two experiment performed. The grouping of blots cropped from different parts of the same gel, or from different gels, is made explicit using a black line delineation the boundary between the gels. **C:** Histogram reporting protein levels of GALNT7 normalized to ß-Actin, in MeT-5A and MPM cell lines The histogram was generated by quantifying blots from two independent experiments and normalizing the intensity of the bands to siCTRL lane. Data are expressed as mean ± SEM. Statistical significance is indicated by asterisk (*), where *=P<0.05; **=P<0.01; ***=P<0.001, compared to siCTRL treatment.

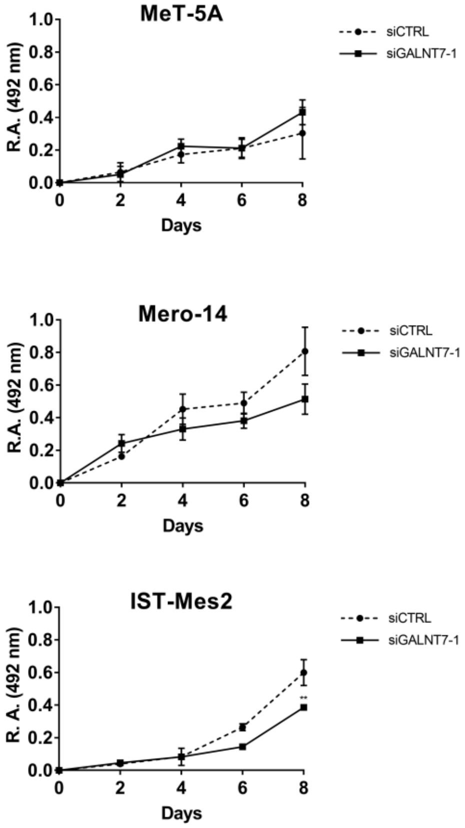

Fig. S11: ***GALNT7*** **silencing and cellular growth.** Proliferation curves of MeT-5A and MPM cells, as Mero-14 and IST-Mes2. The acquisition of optical density, at 492 nm (Relative Absorbance, ”R.A.”), was made every two days after the day of transfection (that is Day 0) with siCTRL or siGALNT7-1. Error bars are SEM from three independent experiments, each performed in triplicate. Statistical significance is indicated by asterisk (*), where *=P<0.05; **=P<0.01; ***=P<0.001, compared to control treatment.

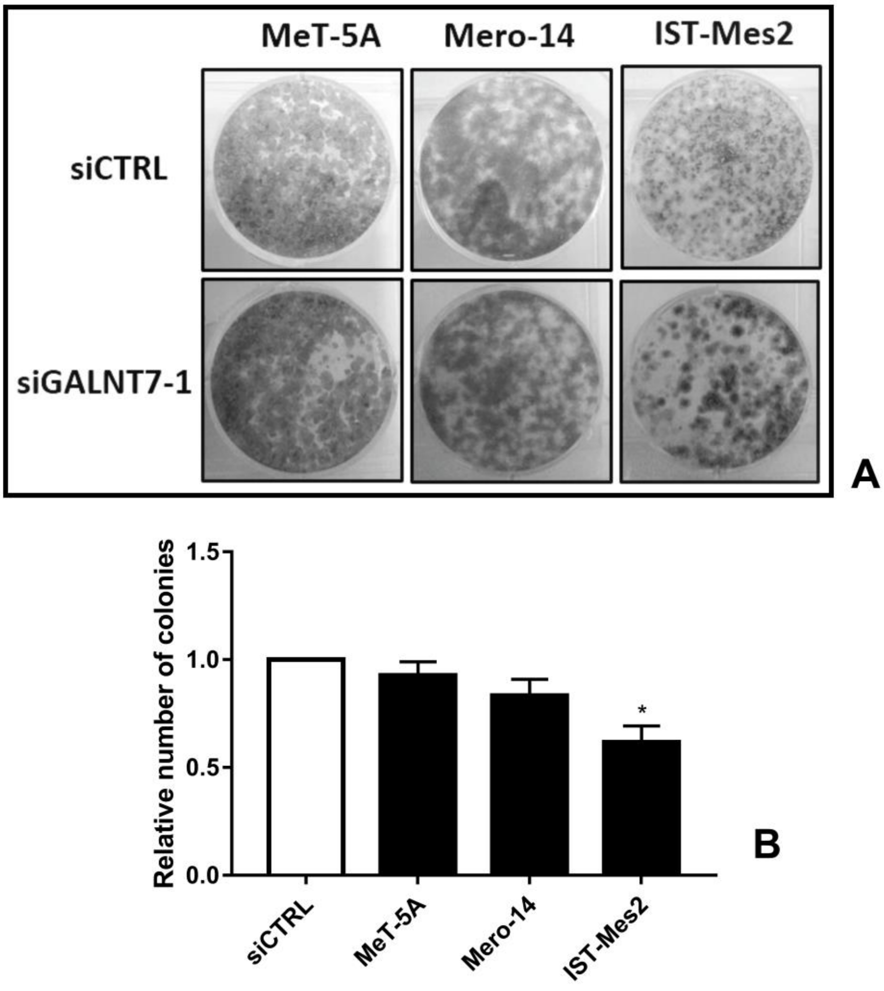

Fig. S12. *GALNT7* silencing and colony formation ability. A: Representative pictures of colonies in MeT-5A and MPM cell lines fixed and stained 14 days after treatment with siCTRL or siGALNT7. This is a representative picture of one experiment. **B:** Histogram represents number of colonies measured 14 days after silencing with siGALNT7-1, compared to siCTRL. Error bars are SEM of three different experiments, each performed in triplicate. Statistical significance is indicated by *, where *=P<0.05; **=P<0.01; ***=P<0.001, compared to control treatment.

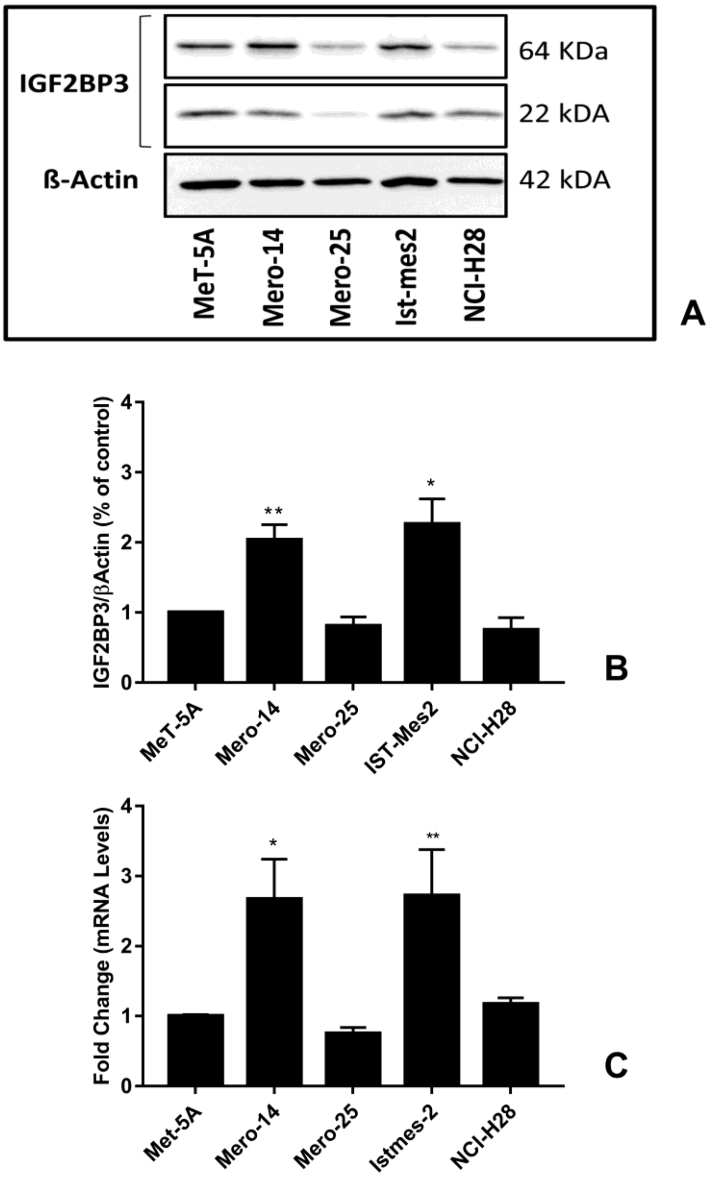

Fig. S13. Basal expression of IGF2BP3 in non-malignant MeT-5A and a panel of MPM cells, as Mero-14, Mero-25, IST-Mes2 and NCI-H28. A: Picture representing protein levels of IGF2BP3, in MeT-5A and mesothelioma cell lines. ß-Actin was used as reference. The present picture is representative of one of two experiment performed. **B:** Histogram reporting protein levels of IGF2BP3, normalized to ß-Actin, in MeT-5A and MPM cell lines. The histogram was generated by quantifying blots from two independent experiments and normalizing the intensity of the bands to MeT-5A lane. Data are expressed as mean ± SEM. **C:** RT-qPCR showing fold changes of mRNA levels of *IGF2BP3*, measured in MPM cell lines and related to MeT-5A, set to one. *RPLP0, HPRT1* and *TPB* were used for normalization. Error bars show the SEM from four independent experiments, each performed in triplicate. Statistical significance is indicated by asterisk (*), where *=P<0.05; **=P<0.01; ***=P<0.001, compared to control MeT-5A cell line.

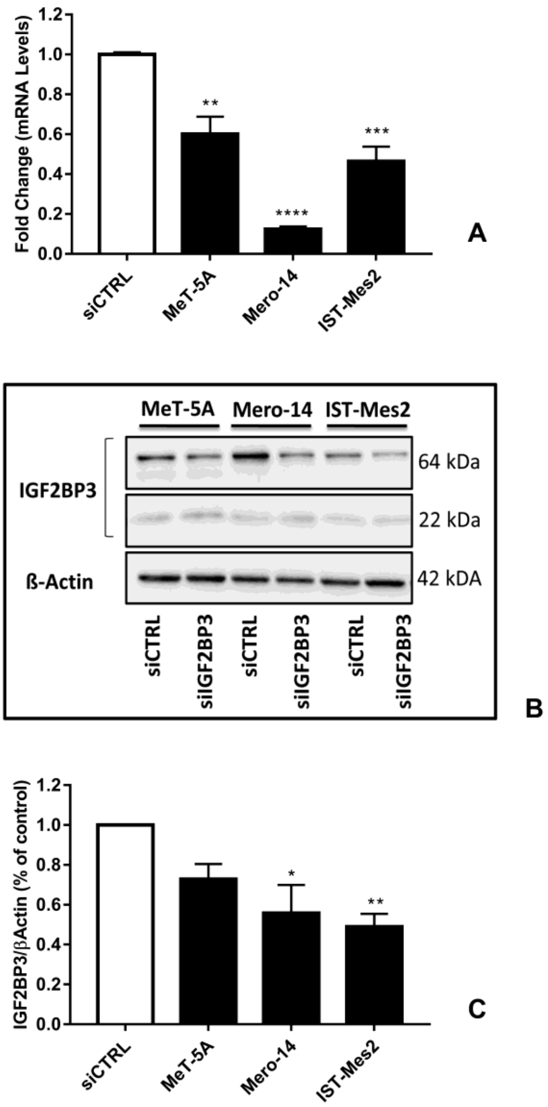

Fig. S14. Silencing of *IGF2BP3* in mesothelial MeT-5A, Mero-14 and IST-Mes2 MPM cell lines. **A:** RT-qPCR showing, as fold change, the mRNA expression levels of *IGF2BP3* in MeT-5A and MPM cells, after treatment with siIGF2BP3-1, related to their own siCTRL. *RPLP0*, *HPRT1* and *TPB* were used for normalization. Error bars are SEM, from three independent experiments, each performed in triplicate. **B:** Picture representing protein levels of IGF2BP3, after its depletion through siIGF2BP3-1, in MeT-5A and MPM cells. ß-Actin was used as reference. The present picture is representative of one of two experiment performed **C:** Histogram reporting protein levels of IGF2BP3 normalized to ß-Actin, in MeT-5A and MPM cell lines. The histogram was generated by quantifying blots from two independent experiments and normalizing the intensity of the bands to siCTRL lane. Data are expressed as mean ± SEM. Statistical significance is indicated by asterisk (*), where *=P<0.05; **=P<0.01; ***=P<0.001, compared to siCTRL treatment.

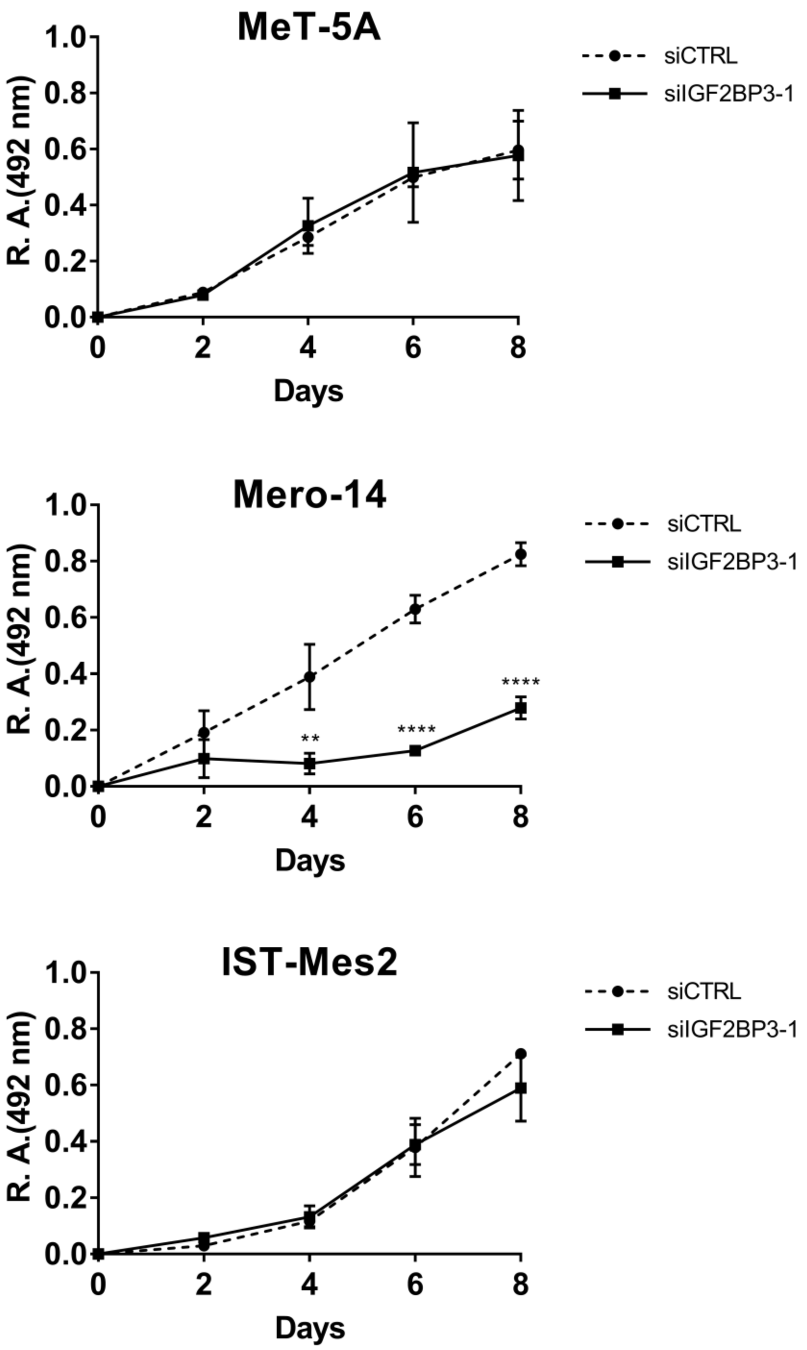

Fig. S15. *IGF2BP3* silencing and cellular growth. Proliferation curves of MeT-5A and MPM cells, as Mero-14 and IST-Mes2. The acquisition of optical density, at 492 nm (Relative absorbance," R.A.") was made every two days after the day of transfection (that is Day 0) with siCTRL or siIGF2BP3-1. Error bars are SEM from three independent experiments, each performed in triplicate. Statistical significance is indicated by *, where *=P<0.05; **=P<0.01; ***=P<0.001, compared to control treatment.

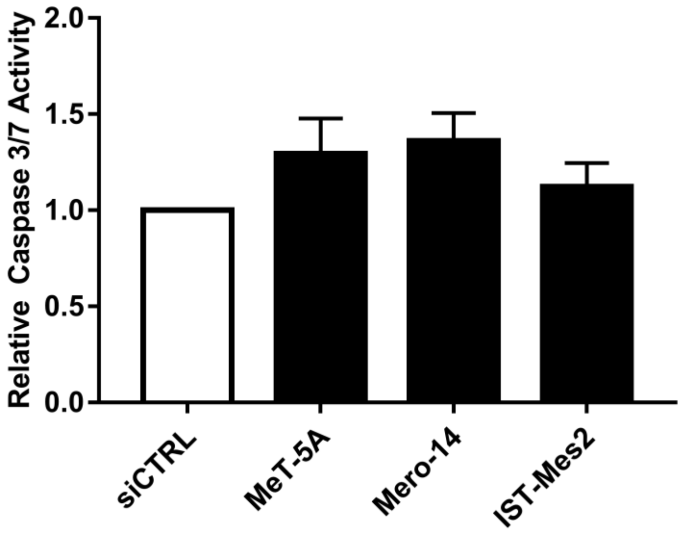

Fig. S16. *IGF2BP3* silencing and caspase -3 and -7 activities. Caspase activity measured in MeT-5A and MPM cells, all transfected with siCTRL (white bar) or siIGF2BP3-1. Error bars are the SEM of three independent experiments, each performed in triplicate. Statistical significance is indicated by *, where *=P<0.05; **=P<0.01; ***=P<0.001, compared to control treatment.

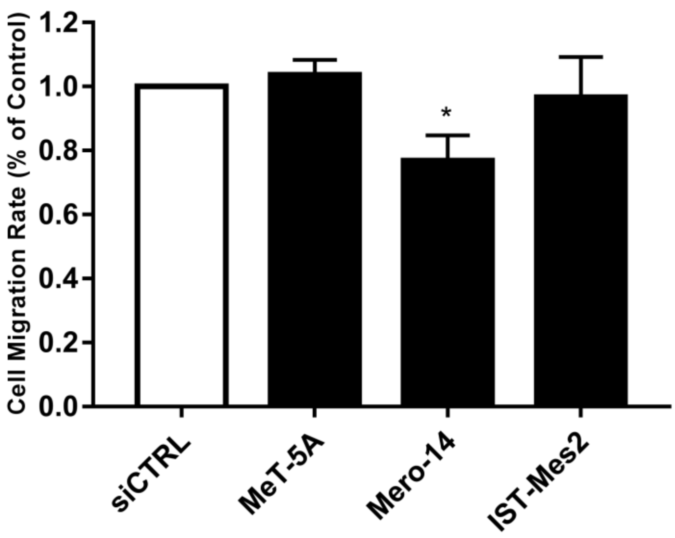

Fig. S17. *IGF2BP3* and migration ability. Changes in cellular migration rate affecting MeT-5A and MPM cell lines, 24h after transfection with siIGF2BP3-1, compared to siCTRL treatment. Error bars are SEM of three different experiments, each performed in triplicate. Statistical significance is indicated by *, where *=P<0.05; **=P<0.01; ***=P<0.001, compared to control treatment.

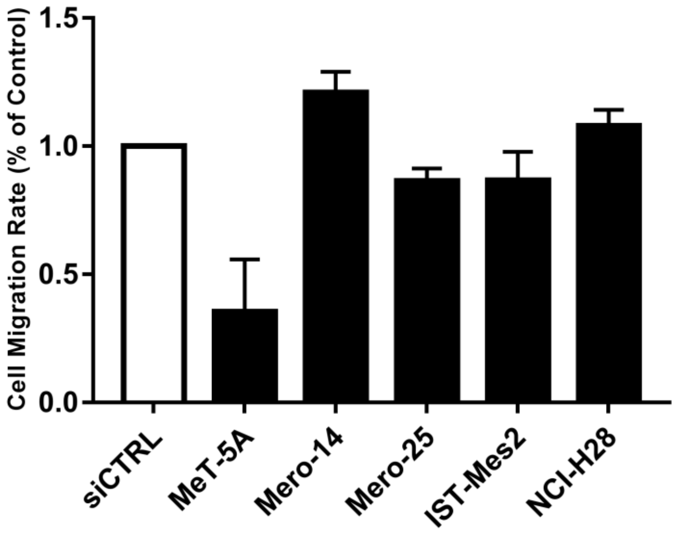

Fig. S18. *RAN* silencing and migration ability. Changes in cellular migration rate affecting MeT-5A and MPM cell lines, 24h after transfection with siCTRL or siRAN-1. Error bars are SEM of three different experiments, each performed in triplicate. Statistical significance is indicated by asterisk (*), where *=P<0.05; **=P<0.01; ***=P<0.001, compared to control treatment.

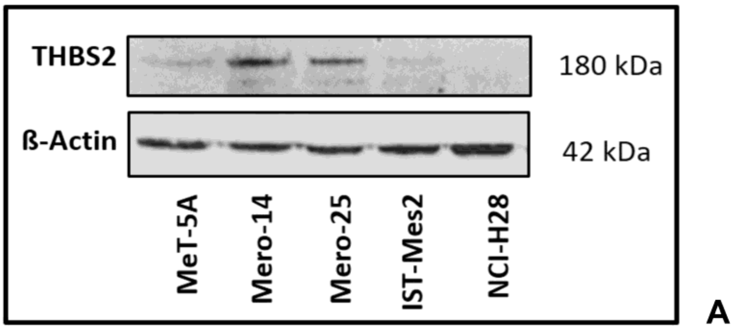

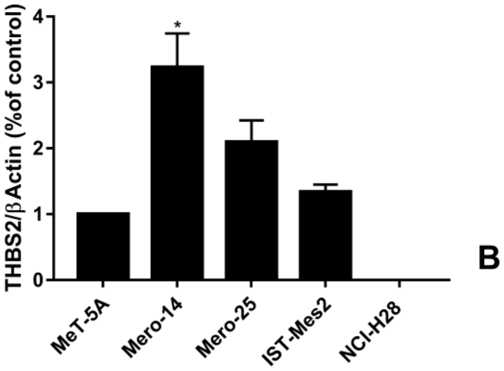

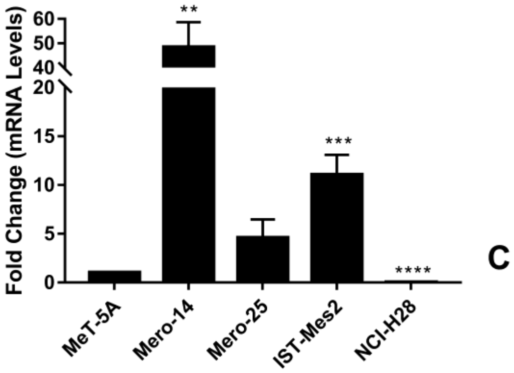

Fig. S19. Basal expression of THBS2 in non-malignant MeT-5A and a panel of MPM cells, as Mero-14, Mero-25, IST-Mes2 and NCI-H28. A: Picture representing protein levels of THBS2, in MeT-5A and mesothelioma cell lines. ß-Actin was used as reference. The present picture is representative of one of two experiment performed. **B:** Histogram reporting protein levels of THBS2, normalized to ß-Actin, in MeT-5A and MPM cell lines. The histogram was generated by quantifying blots from two independent experiments and normalizing the intensity of the bands to MeT-5A lane. Data are expressed as mean ± SEM. **C:** RT-qPCR showing fold changes of mRNA levels of *THBS2*, measured in MPM cell lines and related to MeT-5A, set to one. *RPLP0*, *HPRT1* and *TPB* were used for normalization. Error bars show the SEM from four independent experiments, each performed in triplicate. Statistical significance is indicated by asterisk (*), where *=P<0.05; **=P<0.01; ***=P<0.001, compared to control MeT-5A cell line.

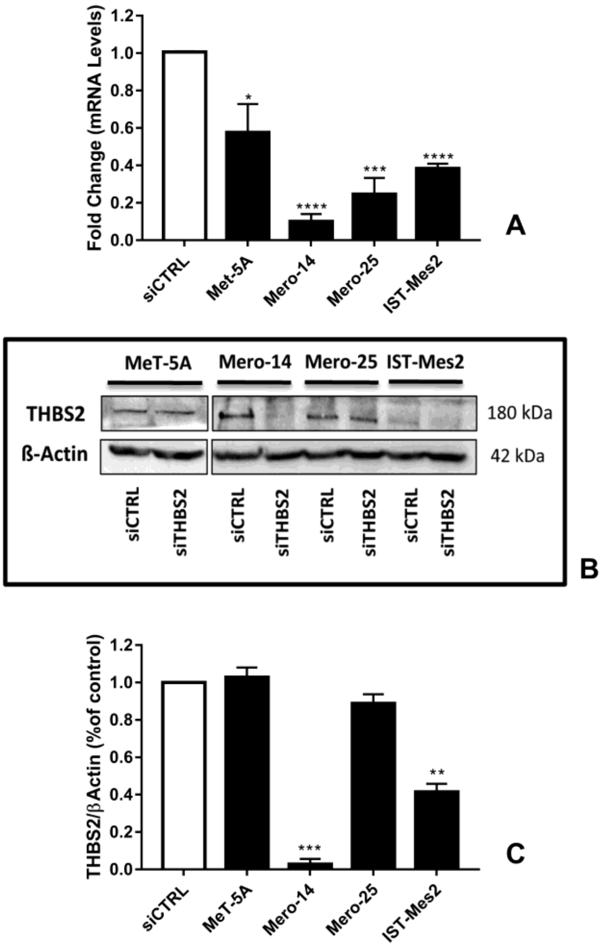

Fig. S20. Silencing of *THBS2* in MeT-5A and MPM cell lines, as Mero-14, Mero-25, IST-Mes2 and NCI-H28. A: RT-qPCR showing, as fold change, the mRNA expression levels of *THBS2* in MeT-5A and MPM cells, after treatment with siTHBS2-1, related to their own siCTRL. *RPLP0*, *HPRT1* and *TPB* were used for normalization. Error bars are SEM, from three independent experiments, each performed in triplicate. **B:** Picture representing protein levels of THBS2, after its depletion through siTHBS2-1, in MeT-5A and MPM cells. ß-Actin was used as reference. The present picture is representative of one of two experiment performed. The grouping of blots cropped from different parts of the same gel, or from different gels, is made explicit using a black line delineation the boundary between the gels. **C:** Histogram reporting protein levels of THBS2 normalized to ß-Actin, in MeT-5A and MPM cell lines. The histogram was generated by quantifying blots from two independent experiments and normalizing the intensity of the bands to siCTRL lane. Data are expressed as mean ± SEM. Statistical significance is indicated by asterisk (*), where *=P<0.05; **=P<0.01; ***=P<0.001, compared to control treatment.

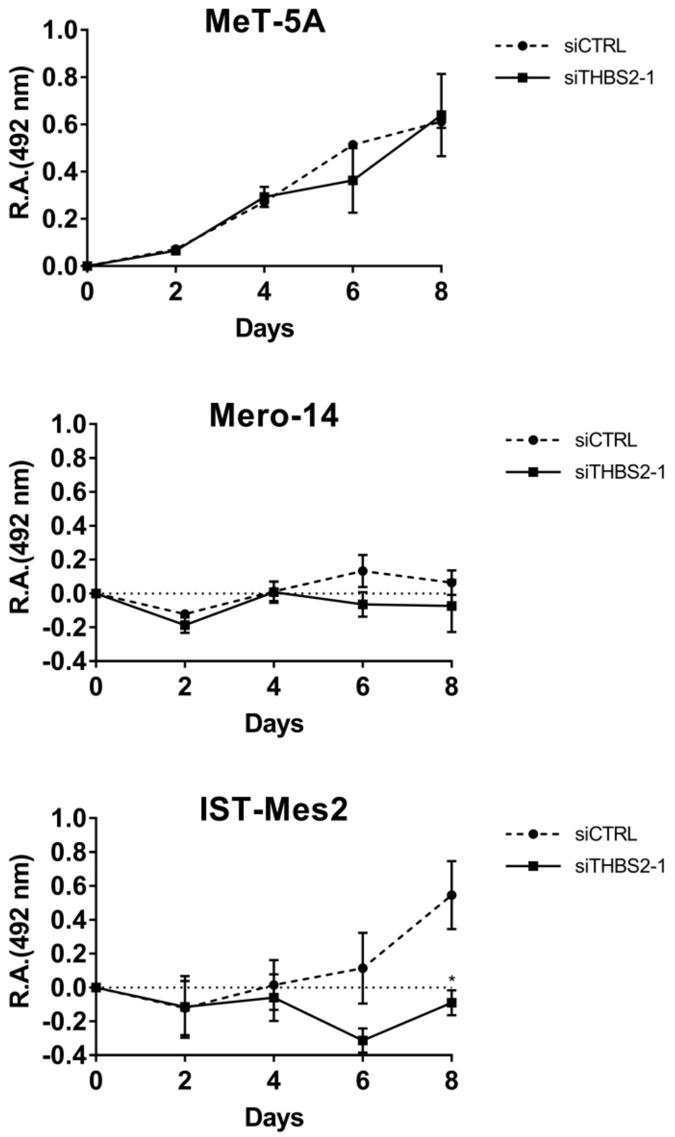

Fig. S21. *THBS2* silencing and cellular growth. Proliferation curves of MeT-5A and MPM cells, as Mero-14 and IST-Mes2. The acquisition of optical density, at 492 nm (Relative absorbance," R.A.") was made every two days after the day of transfection (that is Day 0) with siCTRL or siTHBS2-1. Error bars are SEM from three independent experiments, each performed in triplicate. Statistical significance is indicated by *, where *=P<0.05; **=P<0.01; ***=P<0.001, compared to control treatment.

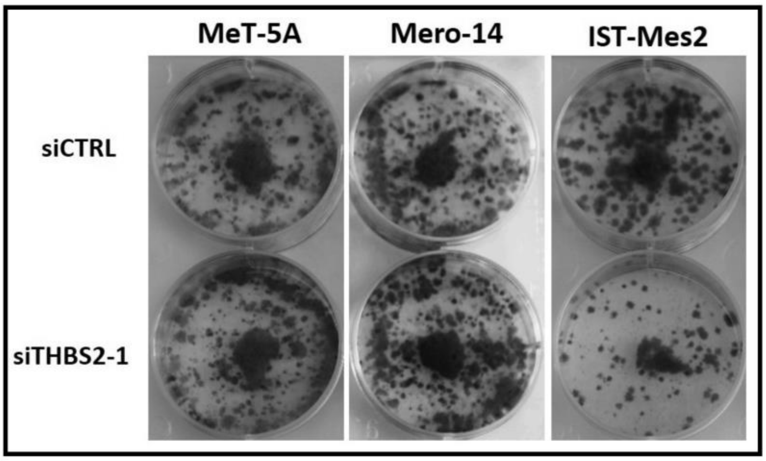

A

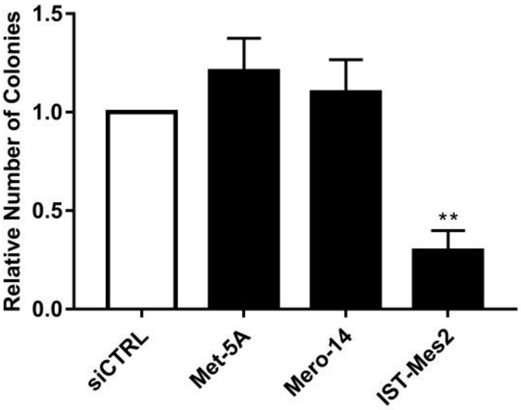

B

Fig. S22. *THBS2* silencing and colony formation ability. A: Representative pictures of colonies in MeT-5A and MPM cell lines fixed and stained 14 days after treatment with siCTRL or siTHBS2-1. **B:** Histogram represents number of colonies measured 14 days after silencing with siTHBS2-1, compared to siCTRL. Error bars are SEM of three different experiments, each performed in triplicate. Statistical significance is indicated by *, where *=P<0.05; **=P<0.01; ***=P<0.001, compared to control treatment.

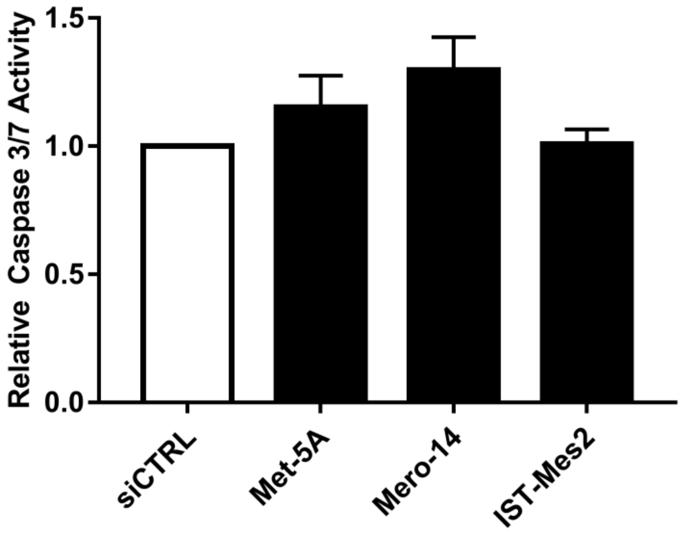

Fig. S23. *THBS2* silencing and caspase -3 and 7- activities. Caspase activity measured in MeT-5A and MPM cells, after transfection with siCTRL (white bar) or siTHBS2-1. Error bars are the SEM of three independent experiments, each performed in triplicate. Statistical significance is indicated by asterisk (*), where *=P<0.05; **=P<0.01; ***=P<0.001, compared to control treatment.
