# Supplementary material for: EIF4G1 and RAN as Possible Drivers for Malignant Pleural Mesothelioma
Source: Int J Mol Sci. 2020 Jul 9;21(14):4856. doi: 10.3390/ijms21144856 (PMC7402288; doi:10.3390/ijms21144856)
Supplement: Supplementary file 1 [file ijms-21-04856-s001.zip › ijms-820510 final supplementary/Original Images_Blots.pdf]

*Article*

# ***EIF4G1* and *RAN* as Possible Drivers for Malignant Pleural Mesothelioma**

**Irene Dell'Anno [1,†], Marcella Barbarino [2,3,†], Elisa Barone [1,†], Antonio Giordano [2,3], Luca Luzzi [4], Maria Bottaro [2], Loredana Migliore [1], Silvia Agostini [1], Alessandra Melani [1], Ombretta Melaiu [1,5], Calogerina Catalano [1,6], Monica Cipollini [1], Roberto Silvestri [1], Alda Corrado [1,7], Federica Gemignani [1,‡] and Stefano Landi [1,*,‡]**

[1] Department of Biology, Genetic Unit, University of Pisa, 56126 Pisa, Italy; irene.dellanno@biologia.unipi.it (I.D.); elisa_barone@ymail.com (E.B.); loredana.migliore@student.unisi.it (L.M.); silvietta.agostini@gmail.com (S.A.); alessandra-29@hotmail.it (A.M.); ombretta.melaiu@unipi.it (O.M.); cal.catalano@gmail.com (C.C.); monica.cipollini@unipi.it (M.C.); r.silvestri17@gmail.com (R.S.); corradoalda@gmail.com (A.C.); federica.gemignani@unipi.it (F.G.)

[2] Department of Medical Biotechnologies, University of Siena, 53100 Siena, Italy; marcella.barbarino@unisi.it (Marcella Barbarino); president@shro.org (A.G.); mariaeusebia.bottaro@gmail.com (Maria Bottaro)

[3] Sbarro Institute for Cancer Research and Molecular Medicine, Center for Biotechnology, College of Science and Technology, Temple University, Philadelphia, PA 19122, USA.

[4] Department of Medicine, Surgery and Neurosciences, Siena University Hospital, 53100 Siena, Italy; dr.luca.luzzi@gmail.com

[5] Immuno-Oncology Laboratory, Department of Paediatric Haematology/Oncology and of Cell and Gene Therapy, Ospedale Pediatrico Bambino Gesù, IRCCS, 00165 Rome, Italy.

[6] Department of Internal Medicine V, University of Heidelberg, 69117 Heidelberg, Germany.

[7] Department of Bioscience, University of Milan, 20133 Milan, Italy.

***** Correspondence: stefano.landi@unipi.it

† The authors contributed equally to this work.

‡ The authors contributed equally to this work

Fig. S1

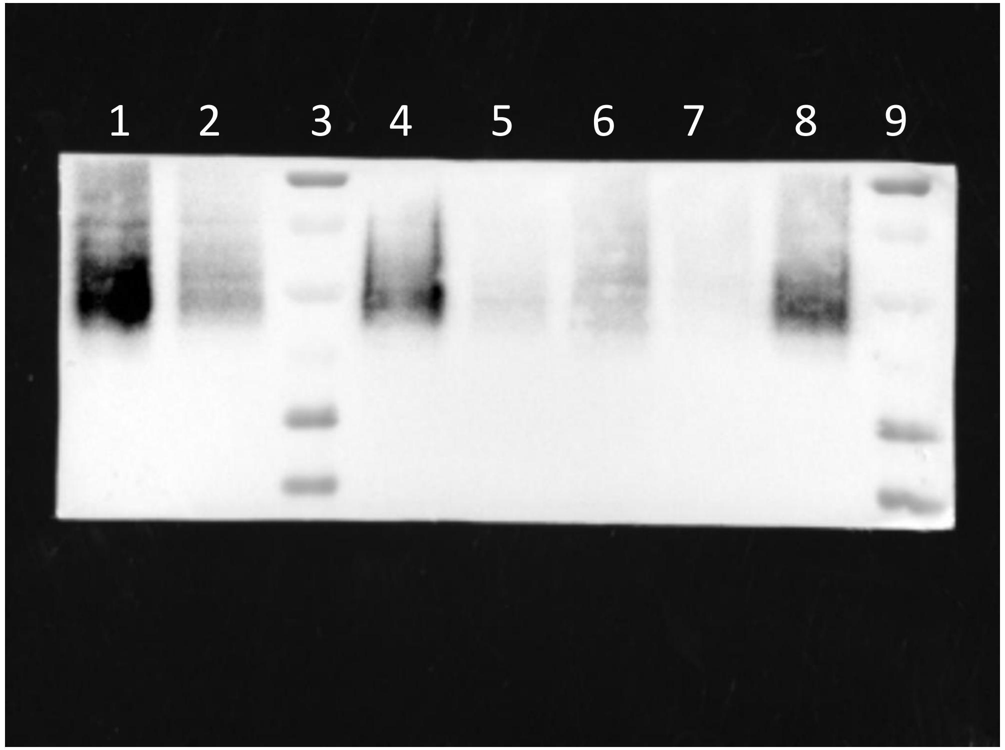


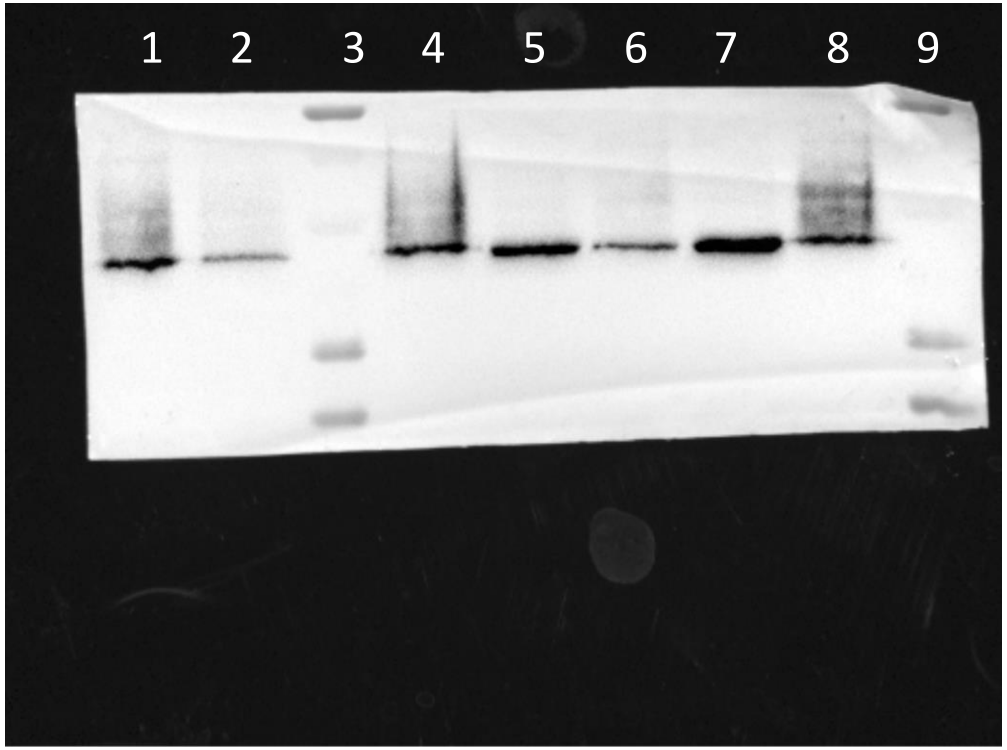


Lane 1: Other sample – TO NOT CONSIDER
Lane 2: Other sample - TO NOT CONSIDER
Lane 3: Ladder
Lane 4: MeT-5A
Lane 5: Mero-14
Lane 6: Mero-25
Lane 7: IST-Mes2
Lane 8: NCI-H28
Lane 9: Ladder

Fig. S2

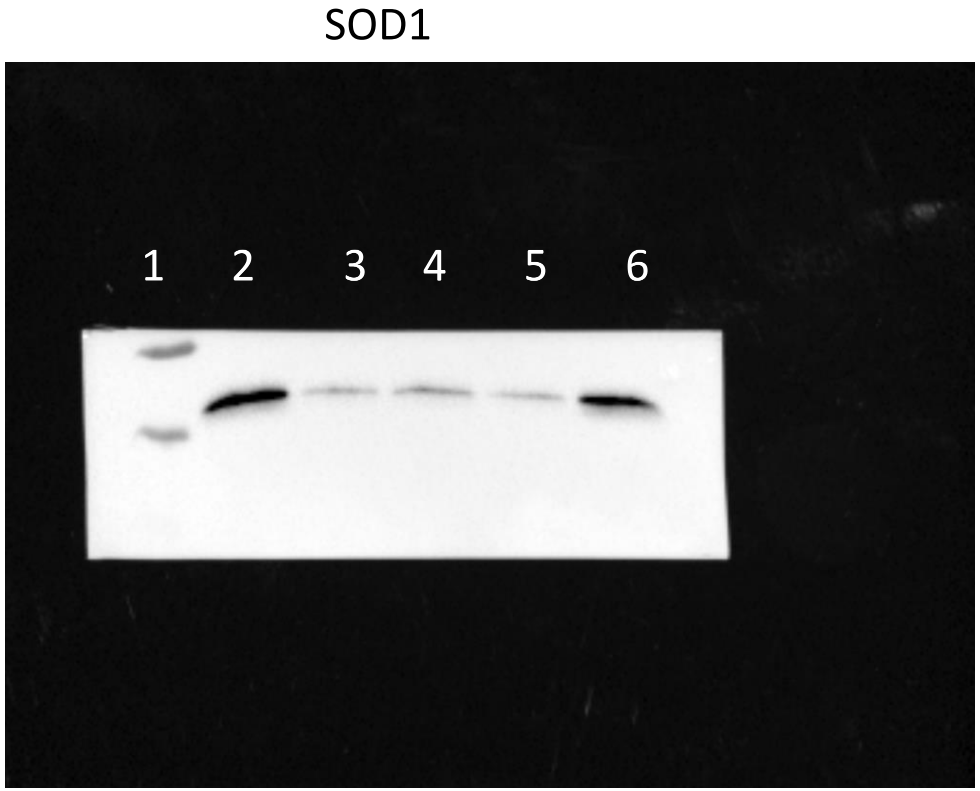


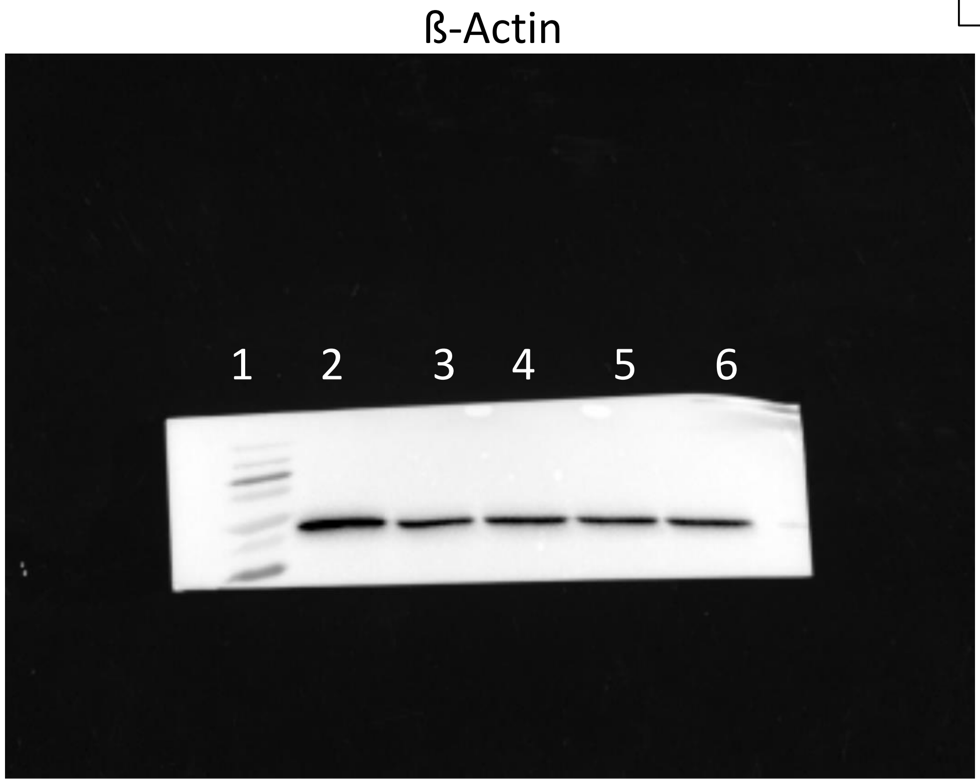


Lane 1:Ladder
Lane 2:MeT-5A
Lane 3:Mero-14
Lane 4:Mero-25
Lane 5:IST-Mes2
Lane 6:NCI-H28

Fig. S3

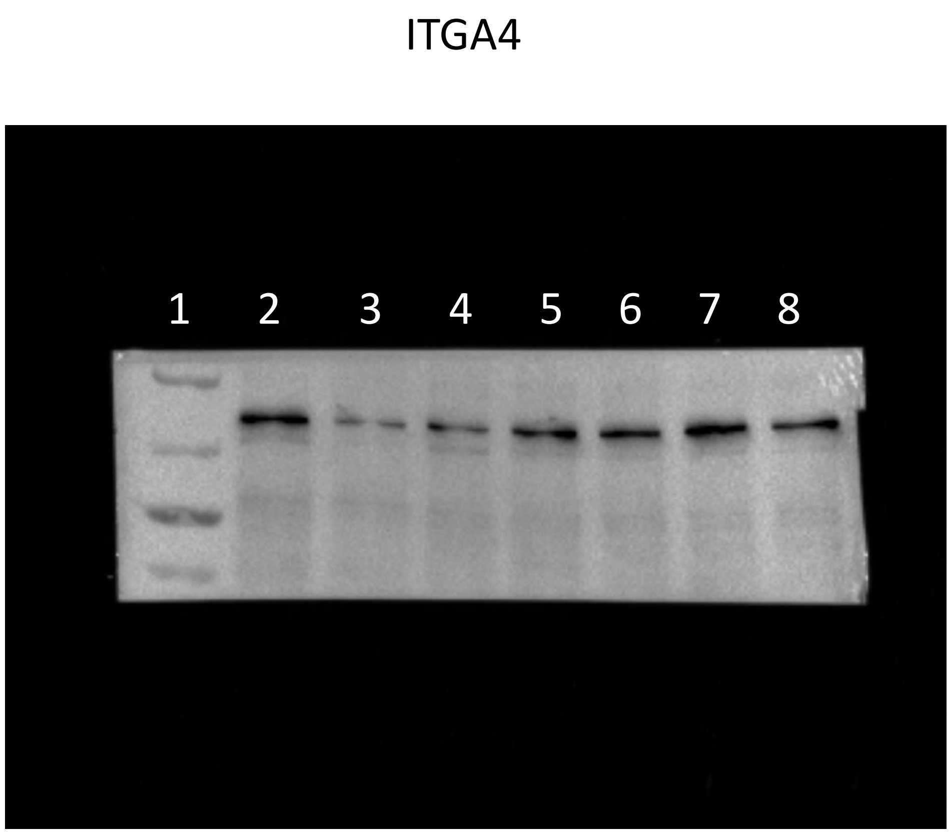


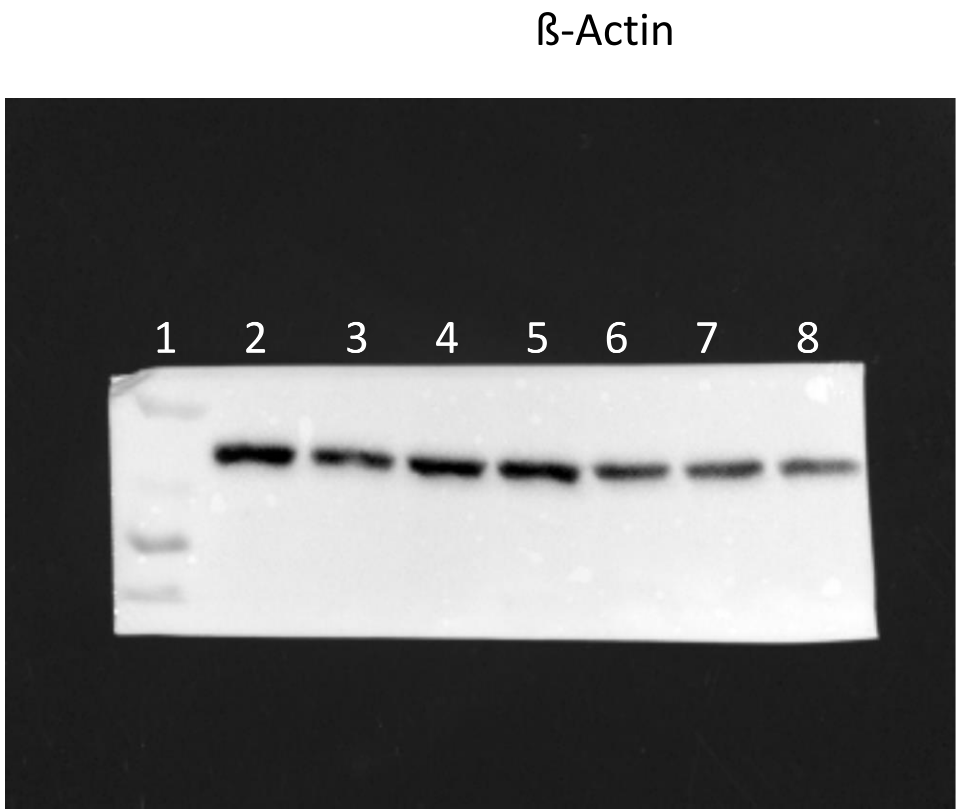


Lane 1:Ladder
Lane 2:MeT-5A
Lane 3:Mero-14
Lane 4:Mero-25
Lane 5:IST-Mes2
Lane 6:NCI-H28
Lane 7: Other sample - TO NOT CONSIDER
Lane 8: Other sample - TO NOT CONSIDER

Fig. S4

ASS1

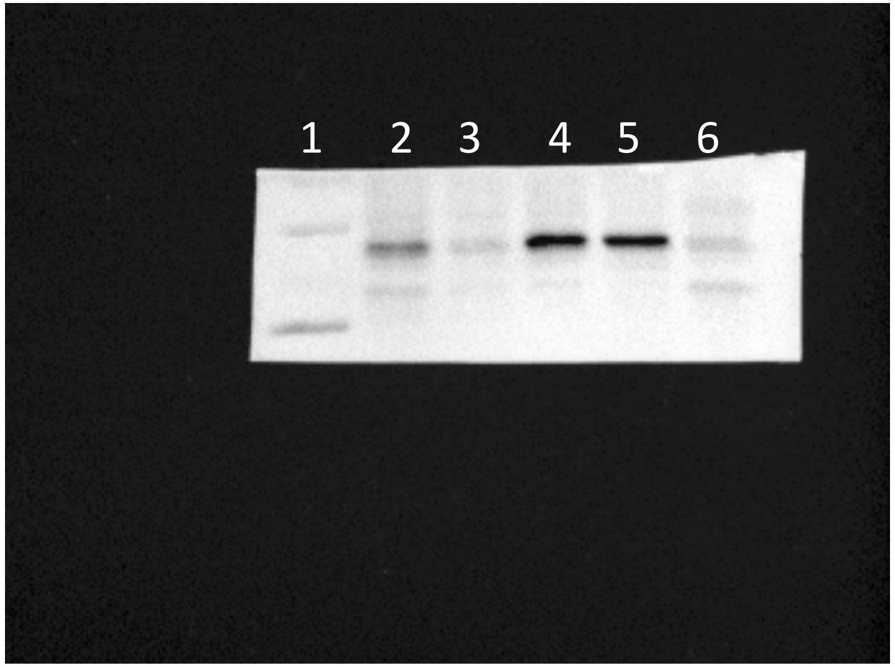


ß-Actin

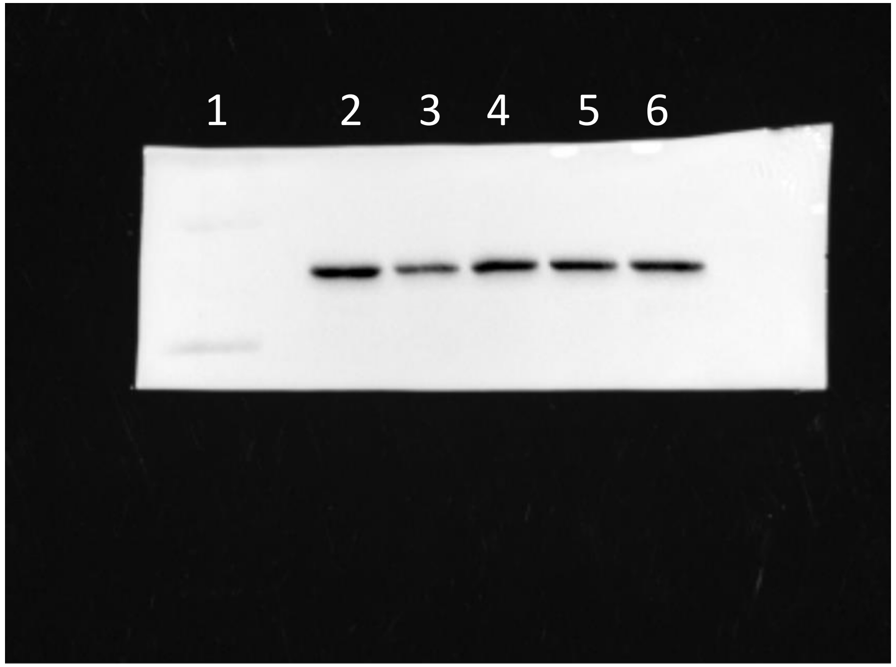


Lane 1:Ladder
Lane 2:MeT-5A
Lane 3:Mero-14
Lane 4:Mero-25
Lane 5:IST-Mes2
Lane 6:NCI-H28

Fig. S5

ASS1

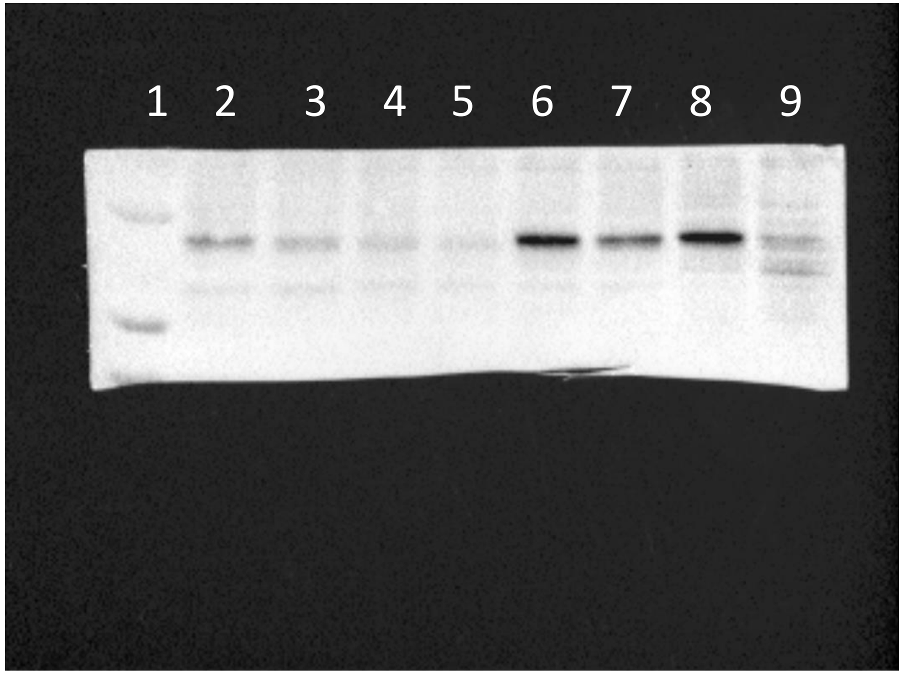


ß-Actin

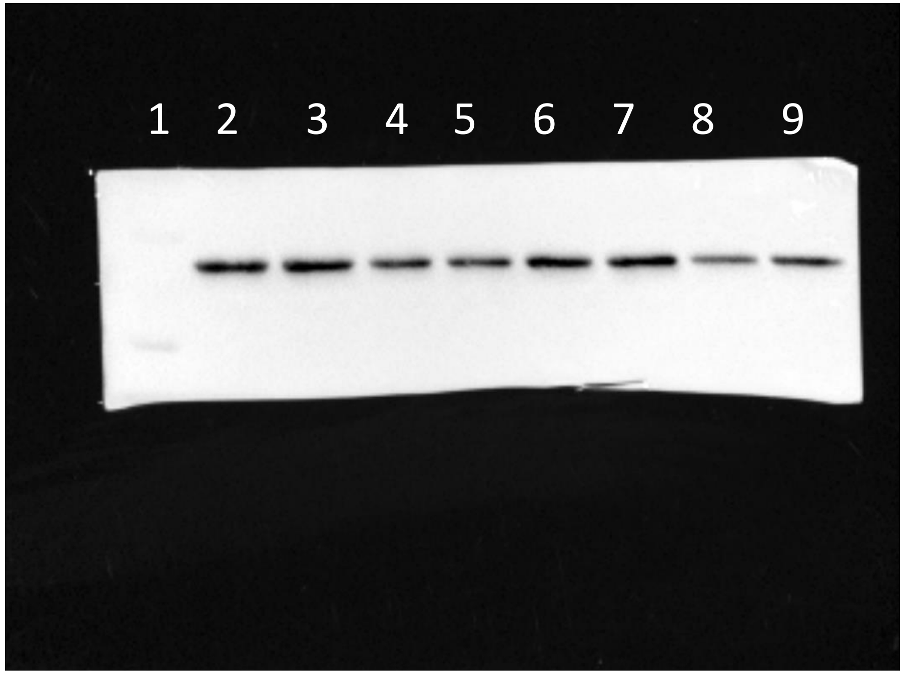


Lane 1:Ladder
Lane 2: MeT-5A siCTRL
Lane 3: MeT-5A siASS1
Lane 4: Other sample - TO NOT CONSIDER
Lane 5: Other sample - TO NOT CONSIDER
Lane 6: Mero-25 siCTRL
Lane 7: Mero-25 siASS1
Lane 8: IST-Mes2 siCTRL
Lane 9: IST-Mes2 siASS1

Fig. 1A

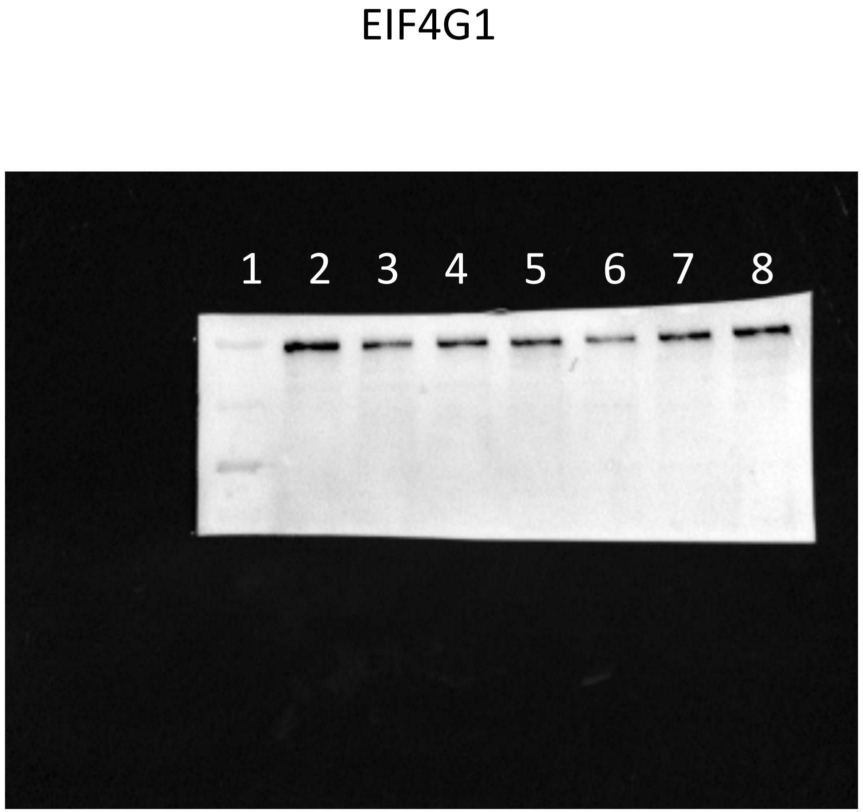


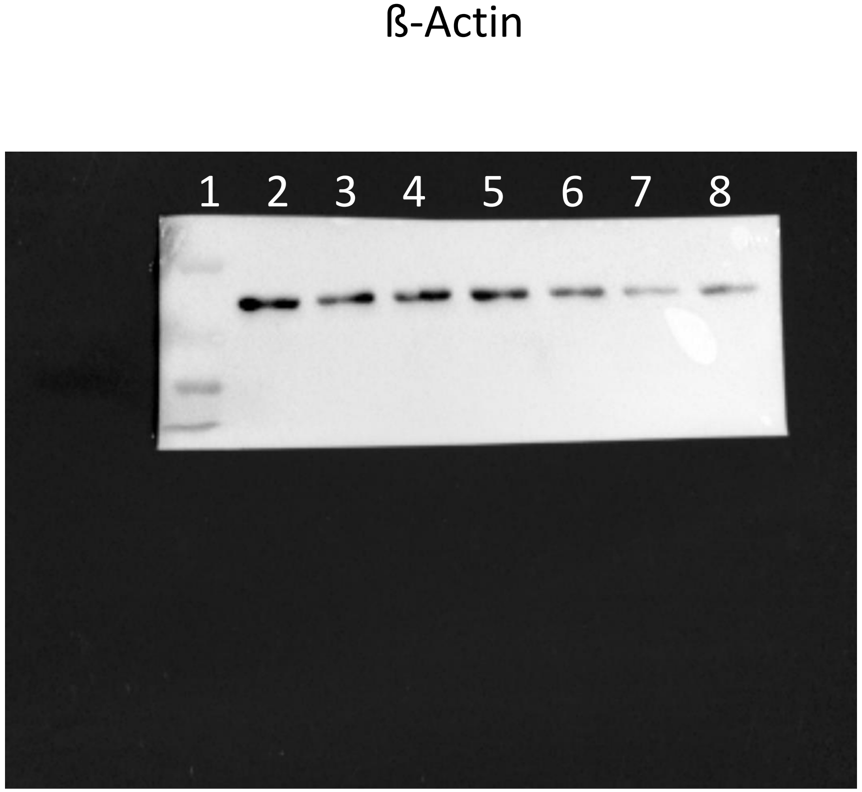


Lane 1:Ladder
Lane 2: MeT-5A
Lane 3: Mero-14
Lane 4: Mero-25
Lane 5: IST-Mes2
Lane 6: NCI-H28
Lane 7: Other sample - TO NOT CONSIDER
Lane 8:Other sample - TO NOT CONSIDER

Fig. 1D

EIF4G1

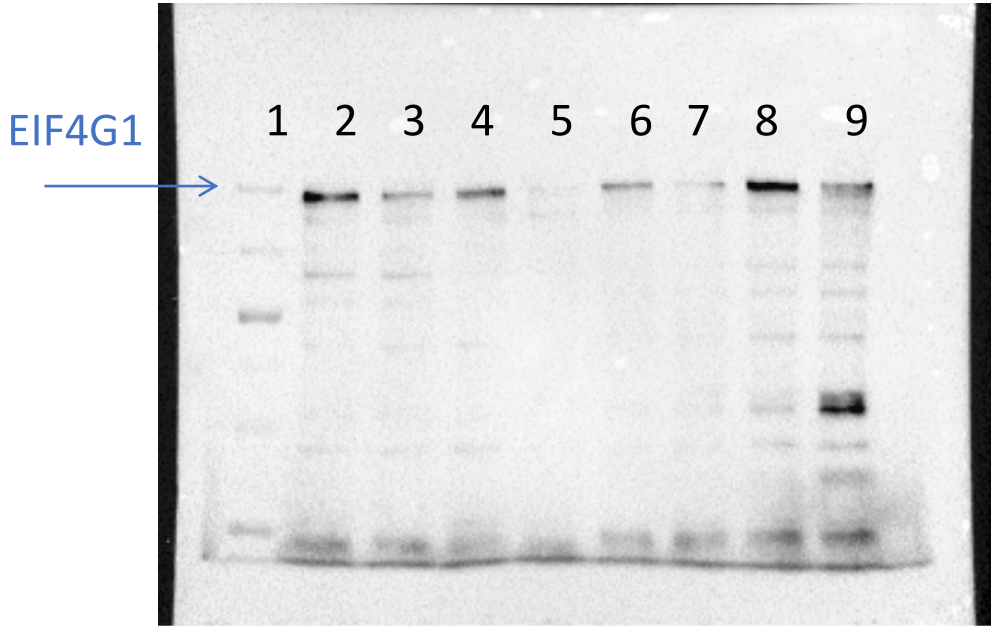


ß-Actin

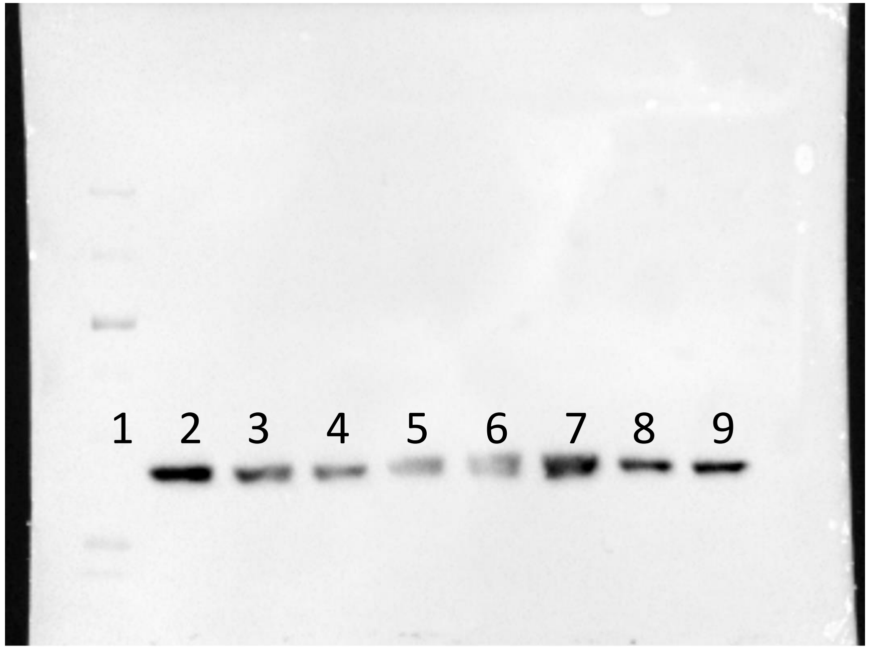


Lane 1:Ladder
Lane 2: Mero-14 siCTRL
Lane 3: Mero-14 siEIF4G1
Lane 4: MeT-5A siCTRL
Lane 5: MeT-5A siEIF4G1
Lane 6: Other sample - TO NOT CONSIDER
Lane 7: Other sample - TO NOT CONSIDER
Lane 8: IST-Mes2 siCTRL
Lane 9: IST-Mes2 siEIF4G1

Fig. S9

GALNT7

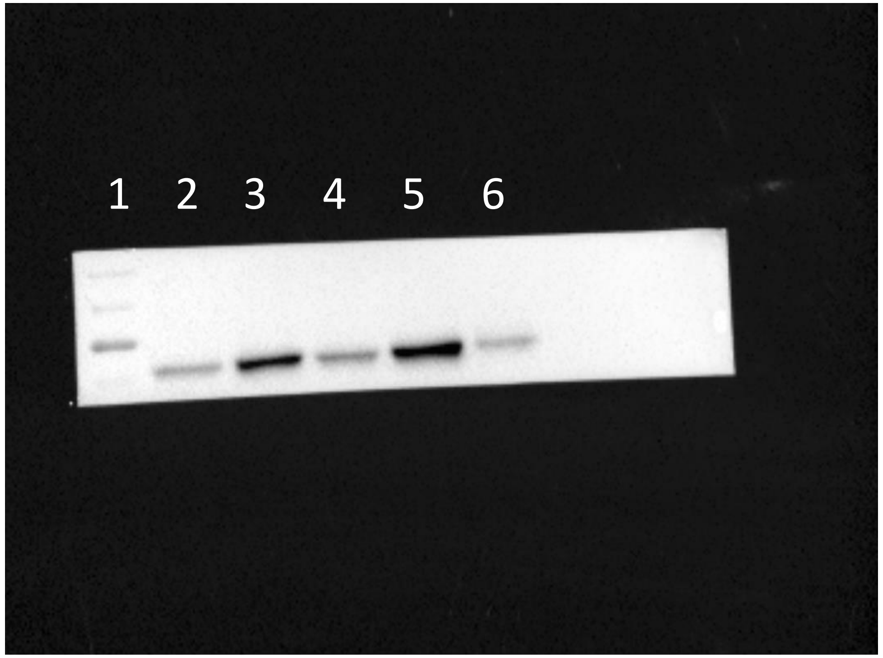


ß-Actin

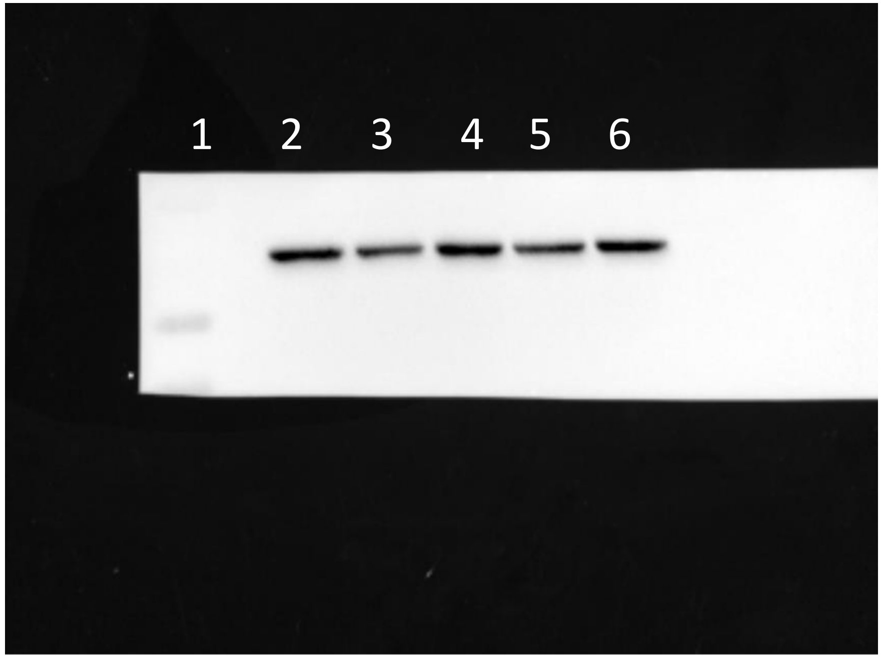


Lane 1:Ladder
Lane 2: MeT-5A
Lane 3: Mero-14
Lane 4: Mero-25
Lane 5: IST-Mes2
Lane 6: NCI-H28

Fig. S10

GALNT7

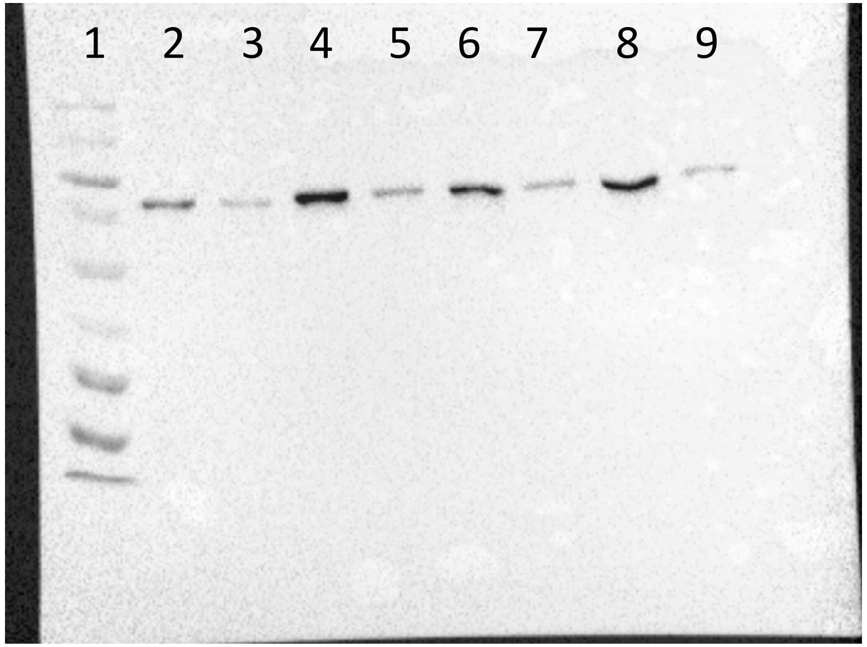


ß-Actin

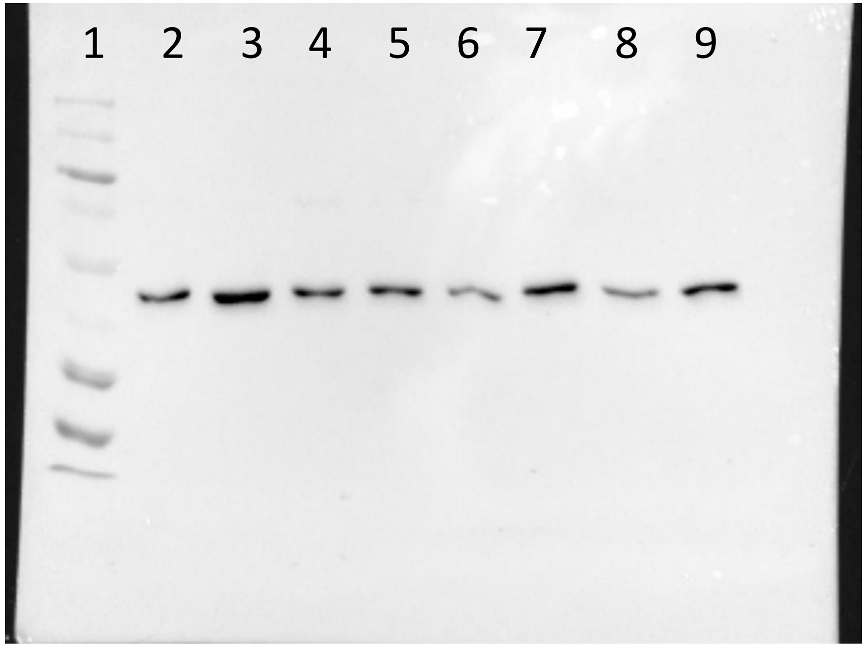


Lane 1:Ladder
Lane 2: MeT-5A siCTRL
Lane 3: MeT-5A siGALNT7
Lane 4: Mero-14 siCTRL
Lane 5: Mero-14 siGALNT7
Lane 6: Other sample - TO NOT CONSIDER
Lane 7: Other sample - TO NOT CONSIDER
Lane 8: IST-Mes2 siCTRL
Lane 9: IST-Mes2 siGALNT7

Fig. S13

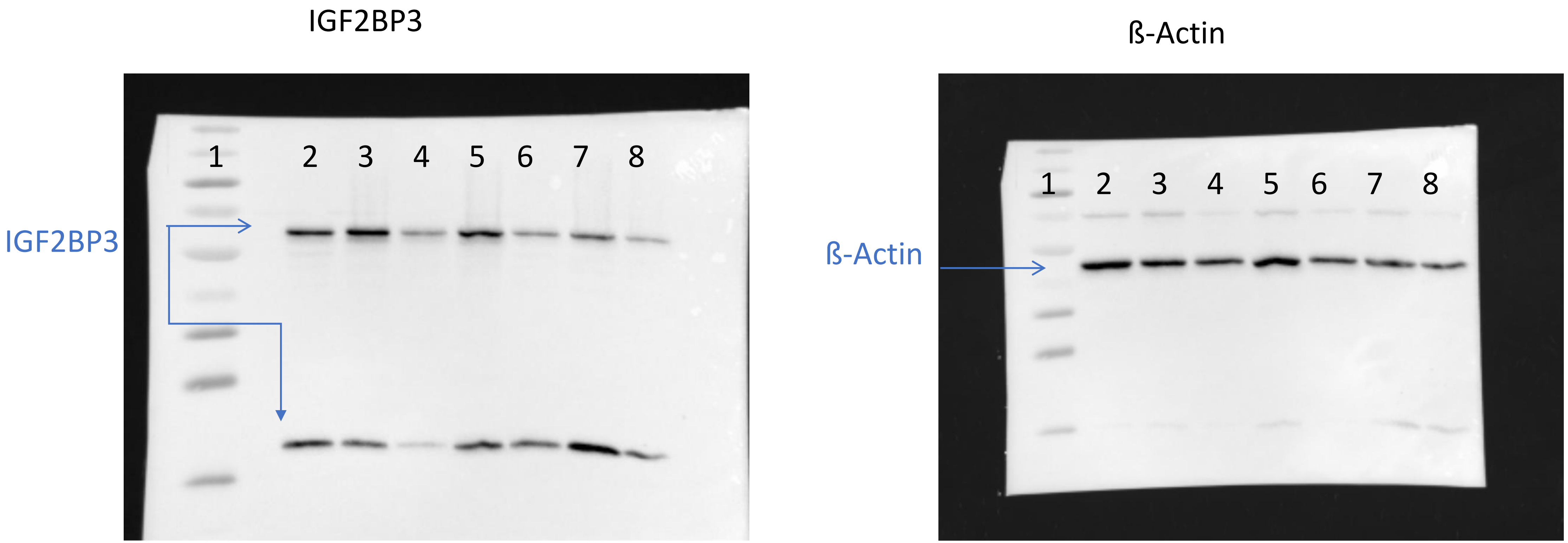


Lane 1:Ladder
Lane 2: MeT-5A
Lane 3: Mero-14
Lane 4: Mero-25
Lane 5: IST-MES2
Lane 6: NCI-H28
Lane 7: Other sample - TO NOT CONSIDER
Lane 8: Other sample - TO NOT CONSIDER

Fig. S14

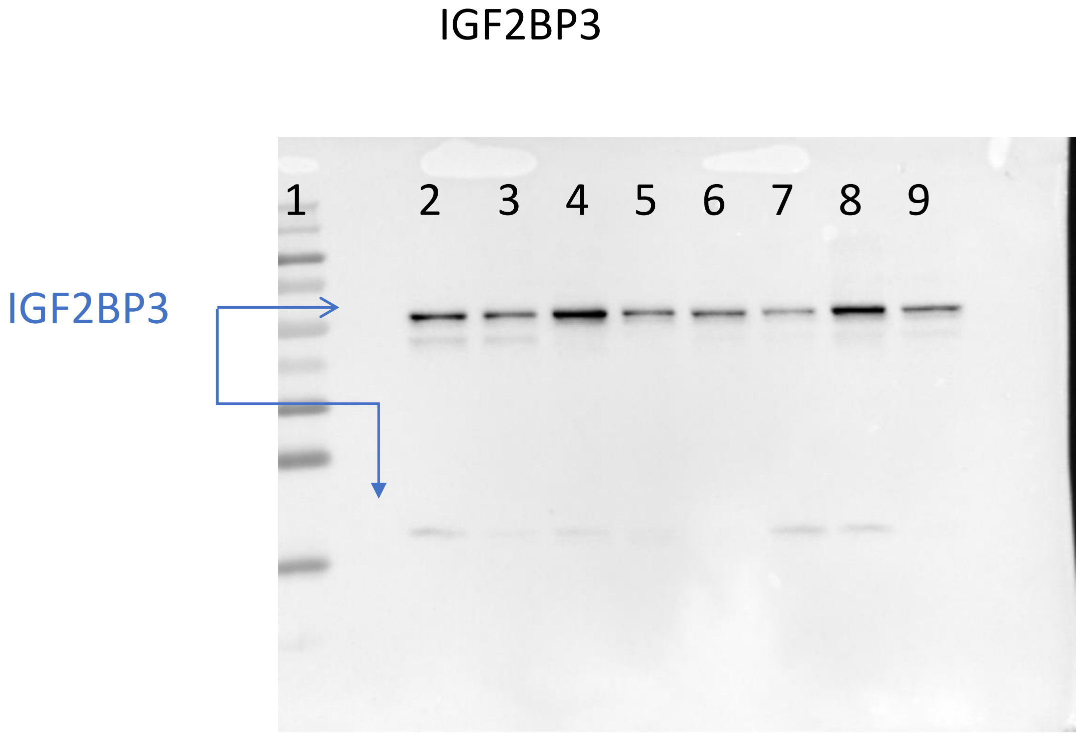


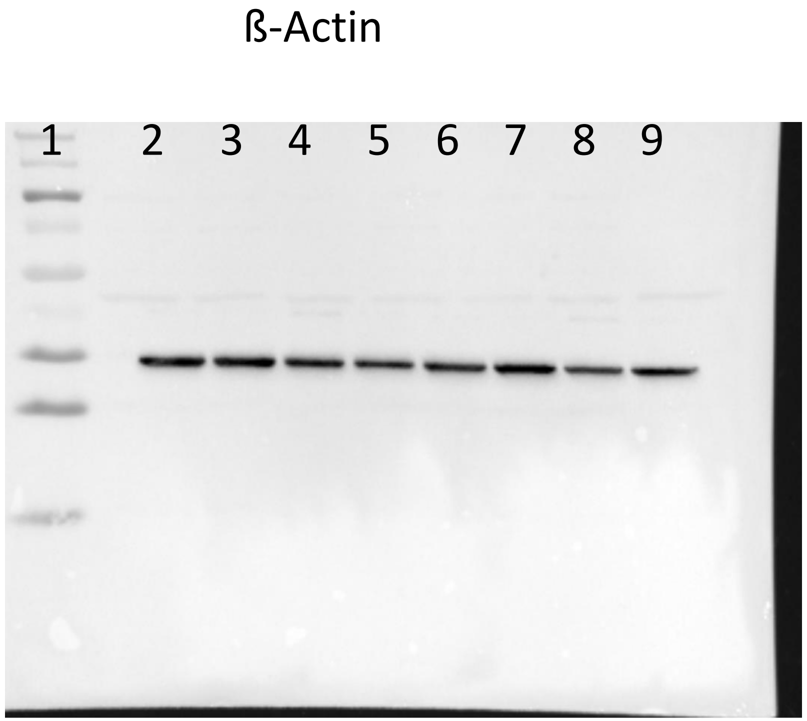


Lane 1:Ladder
Lane 2: MeT-5A siCTRL
Lane 3: MeT-5A siIGF2BP3
Lane 4: Mero-14 siCTRL
Lane 5: Mero-14 siIGF2BP3
Lane 6: IST-Mes2 siCTRL
Lane 7: IST-Mes2 siIGF2BP3
Lane 8: Other sample - TO NOT CONSIDER
Lane 9: Other sample - TO NOT CONSIDER

Fig. 5A

RAN

ß-Actin

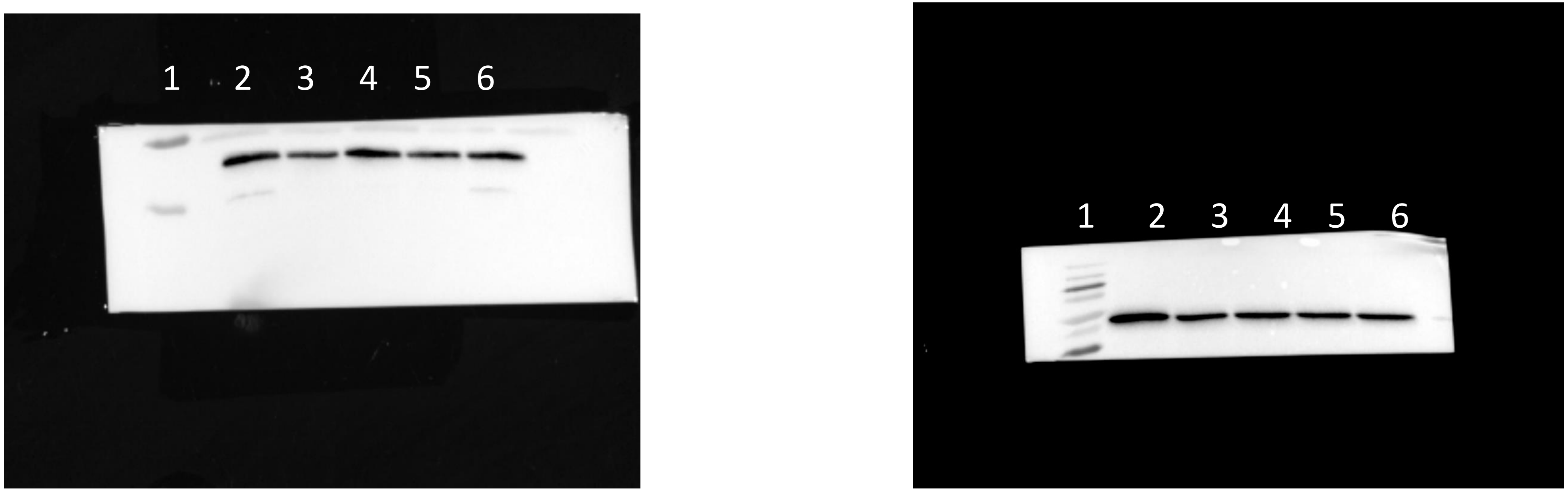


Lane 1:Ladder
Lane 2: MeT-5A
Lane 3:Mero-14
Lane 4:Mero-25
Lane 5:IST-Mes2
Lane 6: NCI-H28

Fig. 5D

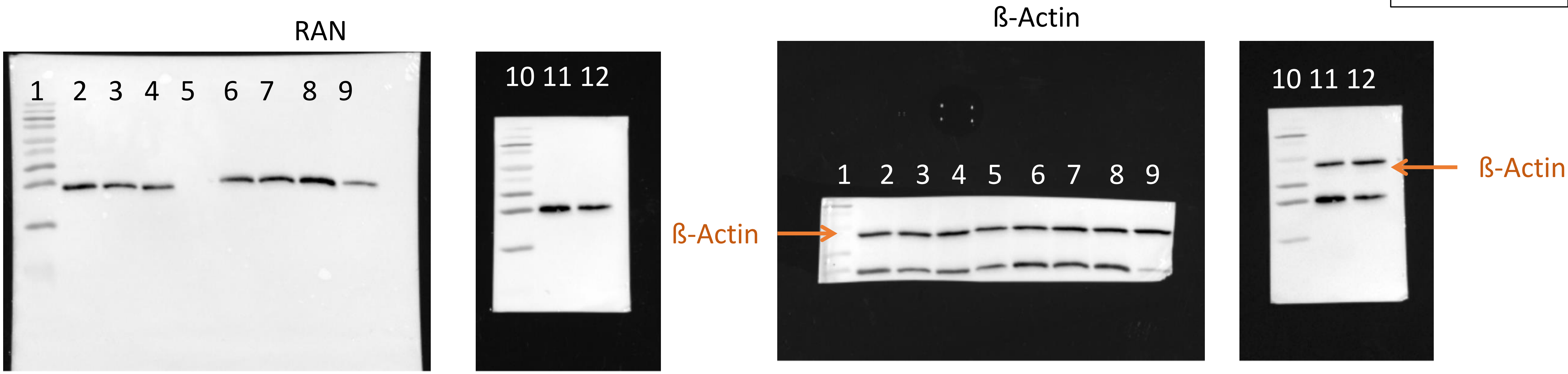


Lane 1:Ladder
Lane 2: MeT-5A siCTRL
Lane 3: MeT-5A siRAN
Lane 4: Mero-14 siCTRL
Lane 5: Mero-14 siRAN
Lane 6: Mero-25 siCTRL
Lane 7: Mero-25 siRAN
Lane 8:IST-Mes2 siCTRL
Lane 9:IST-Mes2 siRAN
Lane 10: Ladder
Lane 11: NCI-H28 siCTRL
Lane 12: NCI-H28 siRAN

Fig. S19

THBS2

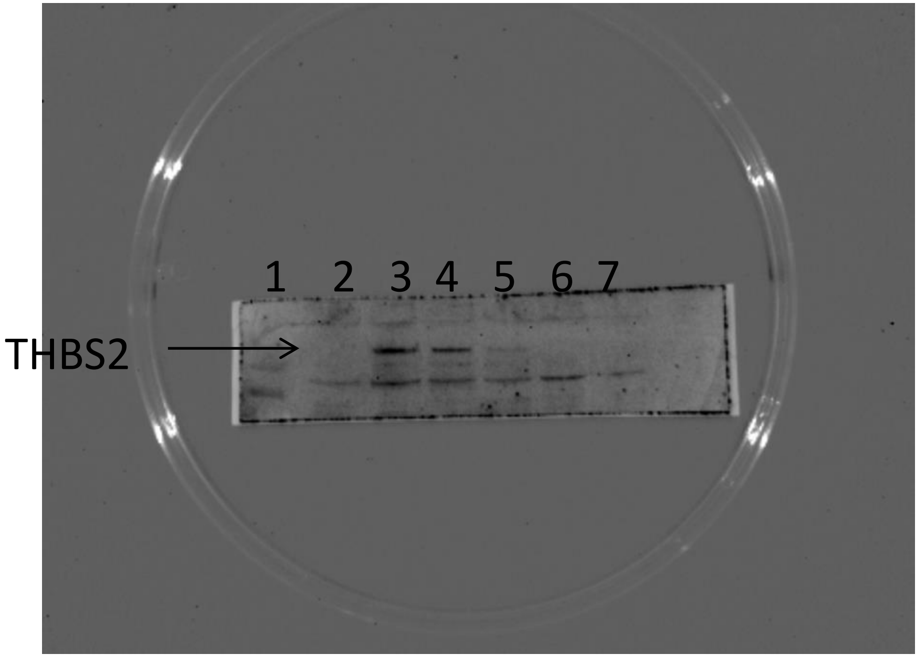


ß-Actin

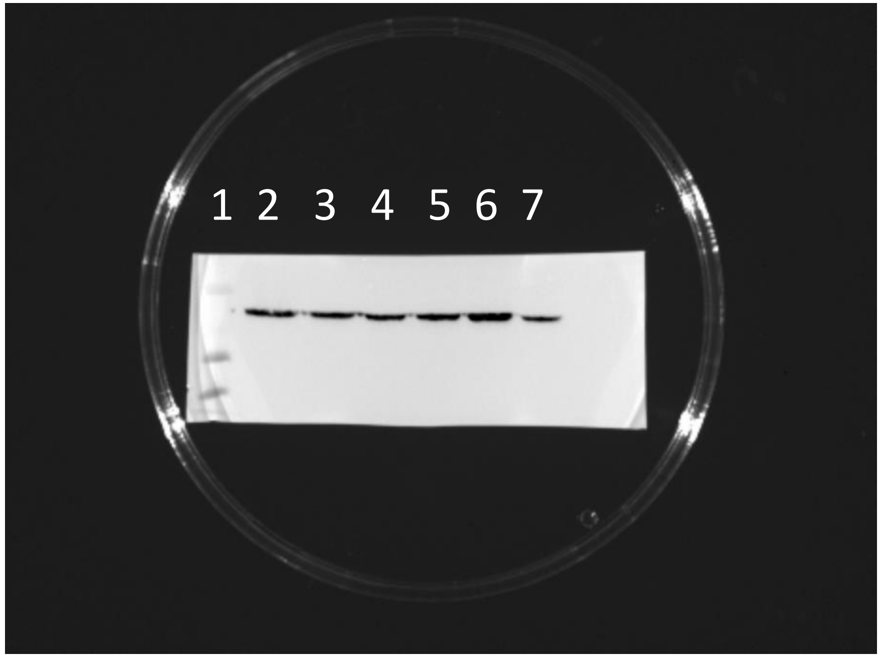


Lane 1:Ladder
Lane 2: MeT-5A
Lane 3:Mero-14
Lane 4:Mero-25
Lane 5:IST-Mes2
Lane 6: NCI-H28
Lane 7: Other sample – TO NOT CONSIDER

Fig. S20

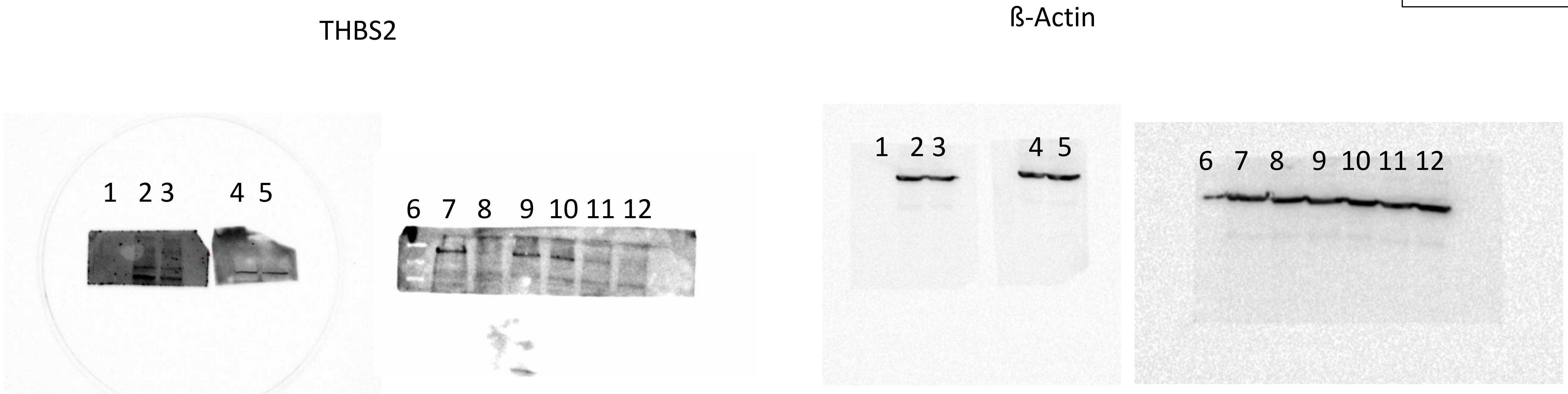


Lane 1:Ladder
Lane 2: Other sample – TO NOT CONSIDER
Lane 3: Other sample – TO NOT CONSIDER
Lane 4: MeT-5A siCTRL
Lane 5: MeT-5A si THBS2
Lane 6: Ladder
Lane 7:Mero-14 siCTRL
Lane 8:Mero-14 siTHBS2
Lane 9:Mero-25 siCTRL
Lane 10: Mero-25 siTHBS2
Lane 11: IST-Mes2 siCTRL
Lane 12:IST-Mes2 siTHBS2
