# Supplementary material for: EIF4G1 and RAN as Possible Drivers for Malignant Pleural Mesothelioma
Source: Int J Mol Sci. 2020 Jul 9;21(14):4856. doi: 10.3390/ijms21144856 (PMC7402288; doi:10.3390/ijms21144856)
Supplement: Supplementary file 1 [file ijms-21-04856-s001.zip › ijms-820510 final supplementary/Supplementary Table 1.pdf]

Supplementary Table 1: Overview of the results for the panel of Cancer Driver Genes (CDGs). Results of silencing (both mRNA and protein levels) and phenotypic assays (proliferation assay, caspase activation, colony formation "CF" assay, and migration assay) are listed for each CDGs. Legend: **X:** experiment not performed; **✓:** Experiment successful; **↑:** the assay shows an increase in the effect; **↓:** the assay shows a decrease in the effect; **=:** the assay shows no effects. Statistical significance is indicated by asterisk (*), where *=P<0.05; **=P<0.01; ***=P<0.001, compared to control treatment.

**CDGs**	**Silencing mRNA/protein**	**Proliferation**	**Caspase activation**	**CF assay**	**Migration assay**
GLUT1	X	X	X	X	X
SOD1	X	X	X	X	X
ITGA4	X	X	X	X	X
ASS1	✓	↓(***)	=	↓(**)	=
EIF4G1	✓	↓(***)	↑(*)	↓(**)	=
GALNT7	✓	↓(**)	=	↓(*)	=
IGF2BP3	✓	↓(***)	=	=	↓(*)
RAN	✓	↓(***)	↑(***)	↓(**)	=
THBS2	✓	↓(*)	=	↓(**)	=
